# Genomic-assisted haplotype analysis and the development of high-throughput SNP markers for salinity tolerance in soybean

**DOI:** 10.1038/srep19199

**Published:** 2016-01-19

**Authors:** Gunvant Patil, Tuyen Do, Tri D. Vuong, Babu Valliyodan, Jeong-Dong Lee, Juhi Chaudhary, J. Grover Shannon, Henry T. Nguyen

**Affiliations:** 1 National Center for Soybean Biotechnology and Division of Plant Sciences, University of Missouri, Columbia, 65211, MO, USA; 2 School of Applied Biosciences, Kyungpook National University, Daegu, 41566, Republic of Korea

## Abstract

Soil salinity is a limiting factor of crop yield. The soybean is sensitive to soil salinity, and a dominant gene, *Glyma03g32900* is primarily responsible for salt-tolerance. The identification of high throughput and robust markers as well as the deployment of salt-tolerant cultivars are effective approaches to minimize yield loss under saline conditions. We utilized high quality (15x) whole-genome resequencing (WGRS) on 106 diverse soybean lines and identified three major structural variants and allelic variation in the promoter and genic regions of the *GmCHX1* gene. The discovery of single nucleotide polymorphisms (SNPs) associated with structural variants facilitated the design of six KASPar assays. Additionally, haplotype analysis and pedigree tracking of 93 U.S. ancestral lines were performed using publically available WGRS datasets. Identified SNP markers were validated, and a strong correlation was observed between the genotype and salt treatment phenotype (leaf scorch, chlorophyll content and Na^+^ accumulation) using a panel of 104 soybean lines and, an interspecific bi-parental population (F_8_) from PI483463 x Hutcheson. These markers precisely identified salt-tolerant/sensitive genotypes (>91%), and different structural-variants (>98%). These SNP assays, supported by accurate phenotyping, haplotype analyses and pedigree tracking information, will accelerate marker-assisted selection programs to enhance the development of salt-tolerant soybean cultivars.

The development of sustainable high-yield varieties that persist under biotic and abiotic stresses is a prerequisite for meeting global food demand^1^. Approximately 20% of the world’s total land area is affected by salt, and this area is increasing as a result of irrigation, water quality and land clearing^2^,^3^. Salinity not only affects plant health but also influences soil function, the soil microbiome, pest/disease infestation and other factors that finally affect overall crop yield.

Soybean [*Glycine max* (L.) Merr.] is considered as a semi-salt sensitive crop^4^, with salinity causing a 20–40% reduction in seed yield^5^,^6^. Salinity occurs when the level of soluble salt – most often sodium chloride (NaCl), is elevated in soil and water. Salt-tolerant plants differ from salt-sensitive ones in their low rate of Na^+^ and Cl^–^ transport to leaves; their ability to compartmentalize salt ions in vacuoles to prevent their build-up in cytoplasm or cell wall, thereby avoiding salt toxicity; and the presence of a salt exclusion mechanism in which Na^+^ and Cl^−^ ions are not stored in roots, ensuring that ions do not accumulate in leaves to a toxic concentration^7^,^8^,^9^,^10^. Soybeans accumulate a higher level of Na^+^ than Cl^−^ in leaves, but the plants more effectively manage to exclude Na^+^ from leaves than Cl^−^ compared with other plants^11^,^12^,^13^. In addition, injury in soybean leaves is more strongly linked (2–3 folds) with Na^+^ than with Cl^−^ concentration^8^. Plants sense salt stress via both ionic (Na^+^) and osmotic stress signals, then activate signal transduction^5^,^14^. Salt-specific symptoms occur mainly in older leaves, where salt transported via the transpiration stream accumulates to higher levels over time^12^. Older leaves no longer expand and therefore newly arriving and previously accumulated salt does not get diluted compared with younger leaves, causing severe leaf chlorosis (i.e. reduced chlorophyll content), and necrosis (i.e. leaf scorching), leading to plant death^12^,^15^,^16^. The underlying physiological mechanism of salinity response has been studied in higher and lower plants^17^,^18^,^19^. It has been proposed that salts outside of roots had an immediate effect on cell growth and associated metabolism and that salt then slowly accumulates inside plants before affecting overall plant function^12^.

Soybeans in the USDA germplasm collection range widely in their reaction to salts^8^. In several early studies, quantitative trait loci (QTL) for soybean salinity tolerance were widely detected and consistently mapped on chromosome (Chr.) 3 (LG *N*)^15^,^20^,^21^,^22^,^23^,^24^. Recently, Qi *et al.*^25^ utilized *de novo* assembly and re-sequencing of a recombinant inbred line (RIL) population to identify the salt tolerance gene *CHX1* (*Glysoja01g005509*/*Glyma03g32900*) in a wild soybean [*Glycine soja* (Siebold & Zucc.)] accession. The identified QTL region overlapped with the previously identified *Ncl* (Chr. 3) locus from cultivar S-100^15^. Hereafter, in this study we refer to *Glyma03g32900* as *GmCHX1*. In another study, using map based cloning and expression profiling, Guan *et al.*^24^ identified the same gene named *GmSALT3* (*Glyma03g32900*) in the commercial cultivated soybean (*G. max*) Tiefeng 8. Compared with salt-sensitive Williams 82 (W82, the reference soybean genome), *GmCHX1* contains a ~3.8 Kb Ty1/copia retrotransposon insertion in exon 3, resulting in a non-functional truncated transcript^25^. Another variant without the retrotransposon produces a full transcript and exhibits a tolerant phenotype. Other than Ty1/copia retrotransposon insertion, sequence variation in the promoter and gene regions of *GmCHX1* also results in salt-sensitive and -tolerant genotypes^25^.

Molecular markers are powerful genomic tools for increasing the efficiency and precision of breeding practices for crop improvement^26^,^27^,^28^. A number of molecular markers such as simple sequence repeats (SSRs), randomly amplified polymorphic DNA (RAPD), and diversity array technology (DArT) have been used in molecular plant breeding over the last several decades; however, these marker technologies are labor-intensive and time-consuming compared with SNP markers^29^. Advances in next-generation sequencing (NGS) and the availability of sequence information have paved the way for the identification and development of SNP markers for several crop species. SNP markers have gained significant importance in plant genetics and molecular breeding due to their suitability for genetic diversity analysis, abundance in genomes, evolutionary relationships and association with complex phenotypic traits^29^. Additionally, their detection and related assays are amenable to automation and thus are useful for high throughput genotyping. The Kompetitive allele-specific polymerase chain reaction (KASPar) assay has emerged as a cost-effective marker assay, especially for molecular breeding applications^30^,^31^ and has been applied in several plant systems^32^,^33^,^34^,^35^ including soybean^36^,^37^. The KASPar assay is specific to a targeted SNP or Indel and consists of two competitive, allele-specific forward primers (labelled with fluorescent dye) and one common primer (http://www.lgcgroup.com/). The identification of molecular markers associated with salinity would be helpful for developing tolerant varieties, especially because phenotyping soybeans according to salinity response is time-consuming, labor-intensive and costly. Additionally, evaluation of salinity-tolerance via conventional breeding programs is difficult because the salt concentration graded in a horizontal or vertical direction in the field^4^. In this study, we evaluated a salt-tolerance locus to verify the allelic variation in 106 soybean resequencing lines and identified SNPs in the promoter as well as exonic and intronic regions for the development of a panel of breeder-friendly KASPar assays.

## Results

### Greenhouse screening for salinity tolerance

**Figure 1:**
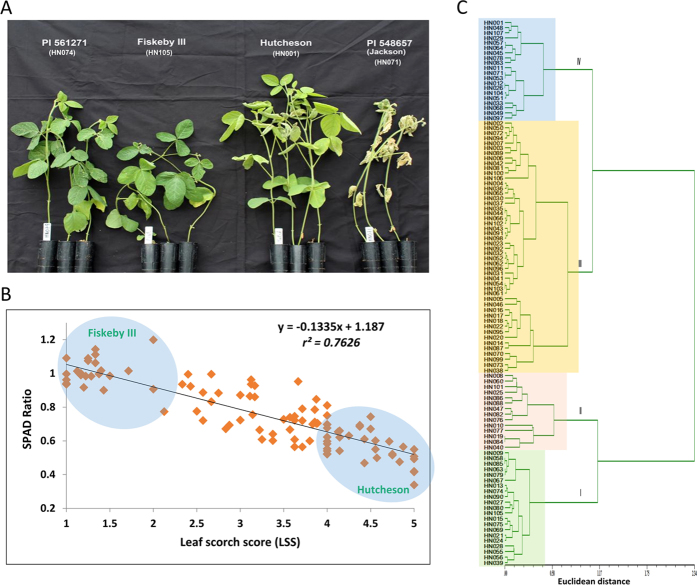
The phenotypic evaluation for salinity tolerance. (**A**) Effect of salt treatment (120 mM NaCl) on four diverse genotypes after 1 week of treatment under greenhouse conditions. Fiskeby III (HN105, salt-tolerant control), Hutcheson (HN001, salt-sensitive control) along with PI 561271 (HN074) and PI 548657 (HN071) showed a high level of tolerance and sensitivity out of 104 germplasm lines tested, respectively. (**B**) Correlation coefficients of leaf scorch score (LSS) and SPAD ratio were calculated from 104 soybean genotypes evaluated for salt tolerance. The highlighted circle shows the most tolerant and sensitive genotypes. (**C**) Dendrogram showing phenotypic (LSS and SPAD ratio) variability relationship between 104 soybean accessions based on phenotypic data. The Euclidean distance (horizontal axis) between objects is used as the distance measure; the clustering was performed using NTSYS software.

To determine salt stress response, 104 out of a total of 106 soybean genotypes with available whole genome sequence data were screened by the Plastic Cone-tainer (PC) method under greenhouse conditions^38^. Salt screening in a greenhouse can be easily controlled and monitored^39^. The reaction of the salt-tolerant (Fiskeby III, HN105) and sensitive (Hutcheson, HN101) genotypes to salt treatment (120 mM NaCl) clearly differed (Fig. 1). The tolerant control scored 1.2 and 0.99, respectively, for the leaf scorch score (LSS) and leaf chlorophyll content (SPAD ratio after and before treatment), while the sensitive control scored 4.8 and 0.52, respectively (Supplementary Table S1). In addition, the tolerant control showed no apparent chlorosis in its leaves, while the sensitive control showed severe chlorosis. Based on a visual rating of the 104 lines evaluated, PI 561271 (HN074) was the most tolerant genotype and PI 548657 (HN071) was the most sensitive genotype (Fig. 1A). A correlation between LSS and SPAD ratio was calculated by regression analysis to determine confidence level. The results indicated a high correlation (*r*^*2*^ = *0.76*) between the two assessment methods (Fig. 1B).

The combination of phenotypic data (SPAD ratio and LSS) was utilized to determine the cluster between salt-sensitive and -tolerant genotypes by Euclidean distance using NTSYS-pc software^40^ (Fig. 1C). The 104 lines were clustered into two major groups; tolerant (35 lines) and sensitive (69 lines). Based on our results, approximately 32% of tested genotypes via re-sequencing were salt-tolerant. A majority of the lines were U.S. cultivars that have been utilized in many soybean breeding programs. The tolerant and sensitive groups were further subdivided into two sub-clusters, I-II and III-IV, respectively. Comparing the mean values of the two clusters (*p* < *0.01*, Duncan’s multiple range test, Table 1) revealed a significant difference. Cluster I showed superior salinity tolerance and had an average LSS of 1.0, whereas cluster IV exhibited higher salt sensitivity and had an average LSS of 4.70. Clusters II and III showed moderate tolerance (LSS = 2.30) and/or moderate sensitivity (LSS = 3.60) in response to salt stress, respectively. Leaf chlorophyll content (SPAD ratio) was significantly decreased from cluster I to cluster IV. The tolerant and sensitive controls were grouped into their respective tolerant and sensitive groups (Fig. 1C). To investigate the accumulation of Na^+^ in leaf tissue, five representative genotypes (3 tolerant and 2 sensitive) were evaluated at 0 mM and 120 mM NaCl concentrations. At 120 mM NaCl, the Na^+^ concentrations in leaf tissue were significantly lower in tolerant genotypes than sensitive genotypes, suggesting a mechanism of limiting Na^+^ transport in leaf tissue (Supplementary Fig. S1). To elucidate the relationship between salt tolerance and allelic variation, whole genome re-sequencing data were used to identify SNP markers as discussed below.

### Genome-wide association study

**Figure 2:**
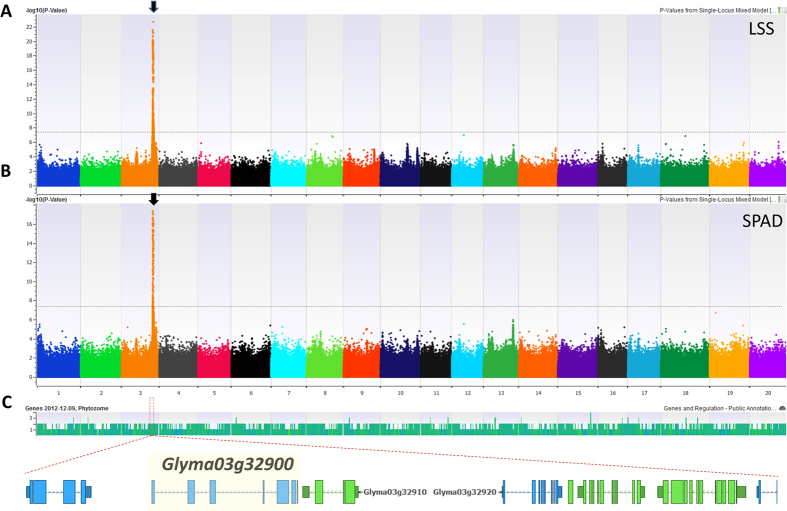
Manhattan plots of GWAS for (**A**) LSS and, (**B**) SPAD ratio, in 106 soybean lines using WGRS dataset. Negative log_10_-transformed *P* values of SNPs from genome-wide scan for salinity traits using EMMAX model including kinship and population structure are plotted against positions on each of the 20 chromosomes; (**C**) genes underlying significant trait-associated SNPs on Chr. 3. The horizontal line denotes the calculated threshold value for declaring significant association.

To identify and obtain insight into genes controlling salt tolerance in soybean, a genome-wide association study (GWAS) was performed on the106 soybean lines using an expedited single-locus mixed model (EMMAX) implemented in the SVS suite (v8.1.5). The efficient EMMAX model corrected for confounding effects due to subpopulation structure and includes PCA-Eigen vectors and identity by descent (IBD) matrices^41^. The WGRS data of 106 lines (Valliyodan *et. al.* unpublished) as well as publically available SoySNP50K^42^ (~42,509) SNP data were utilized for the analysis and comparison between of the datasets. After initial quality filtering, over 5 million SNPs from WGRS data and over 37,400 SNPs generated by SoySNP50K^42^ were considered for association mapping. Using WGRS and SoySNP50K, both datasets pin-pointed a single and highly significant association for average SPAD ratio and LSS (Supplementary Table S1A, B) on Chr. 3 (40520215-40637459) (Fig. 2, Supplementary Fig. 2). This region overlapped with a previously identified salt-tolerant locus, and the gene *Glyma03g32900* (*GmCHX1*), characterized for salt tolerance in soybean, was detected (Fig. 2C). GWAS identified 19 and 11 SNPs using SoySNP50K data, and 401 and 328 SNPs were identified using WGRS data that were associated with LSS and SPAD, respectively, at the significance level –log_10_ *p* ≥ 7.3. The most significant SNP (-log_10_ *p* 22.62) of WGRS data for LSS was identified within the *GmCHX1* gene (fourth intron) and explained 63% of the phenotypic variation (Supplementary Table S1B). Genome-wide analysis showed that natural variation associated with this gene has a major impact on salt tolerance in soybean. Therefore, subsequent analysis was focused on the *GmCHX1* gene.

### Hierarchical clustering using soybean whole genome re-sequencing data

**Figure 3:**
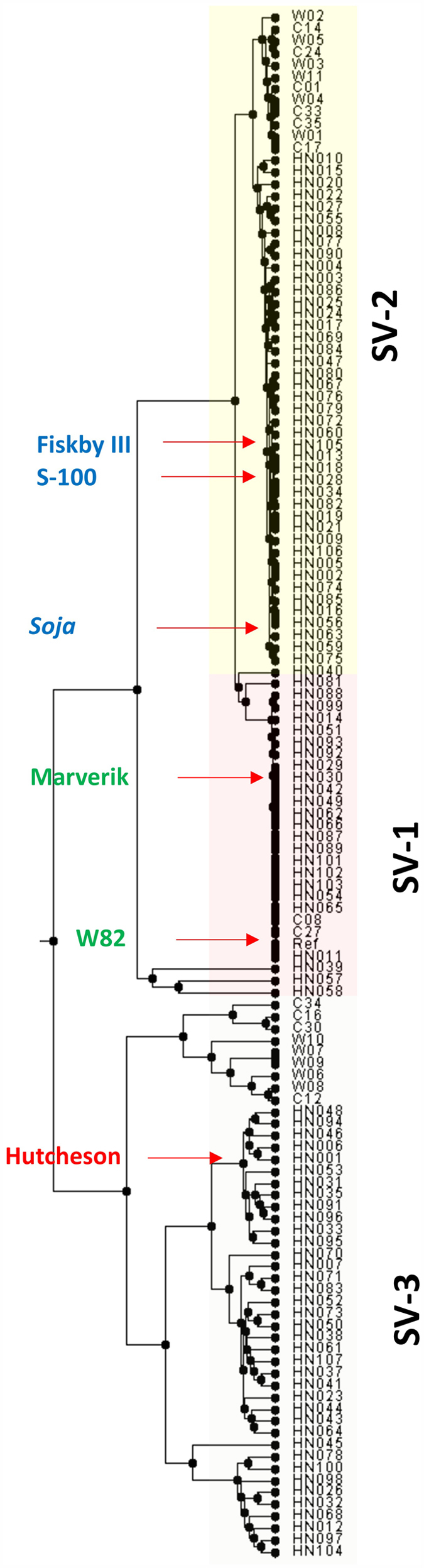
Hierarchical clustering of 129 diverse lines based on SNP information of WGRS data. Clusters were observed in 23 lines^44^ [denoted with ‘C’ and ‘W’] and 106 lines [denoted with ‘HN’]. Lines representing structural variation are highlighted with different colors (see details in Fig. 4A). Lines with known salinity reaction are shown with pointed arrow in each group.

Analysis of the soybean *GmCHX1* gene provided an opportunity to obtain an overview of allelic variation using the soybean whole genome re-sequencing (WGRS) data. The wealth of whole genome resources for soybean provides a unique method to study natural variation in germplasm and further allows the functional characterization of candidate genes^24^,^25^,^43^. Complete genome sequences for the 106 soybean genotypes, sequenced at approximately 15X coverage, were utilized for analysis. To observe phylogenetic clustering, multi-sampled SNPs for the *GmCHX1* locus, including a 2 Kb promoter region (Chr. 3: 40621077-40634451) were extracted from the WGRS data and were utilized to infer phylogenetic relationships. In addition to the 106 lines, we included 23 previously reported salt-tolerant and -sensitive genotypes from 31 soybean re-sequencing lines^44^. These 23 lines were sequenced at relatively lower coverage compared with the other 106 lines and grouped closely together in the phylogenetic tree (Fig. 3). Phylogenetic analysis of 129 lines showed three distinct clusters associated with structural and allelic variation at the *GmCHX1* locus (Fig. 3). Based on the salt-treatment phenotypic data (LSS and SPAD ratio), tolerant indicator lines such as S-100 (HN028) and Fiskeby III (HN105) as well as a wild soybean genotype, PI483463 (HN063) were clustered separately. On the other hand all of the sensitive genotypes were clustered into two distinct subgroups with the known salt-sensitive indicator lines Hutcheson (HN001), W82 (Ref) and Maverick (HN030). In agreement with previous studies^25^, the 23 genotypes from 31 re-sequenced lines were also grouped into their respective salt-tolerant or -sensitive clusters. In addition, we utilized the genome sequence information of 93 U.S. ancestral lines^45^ to explore allelic diversity at the *GmCHX1* locus. These 93 diverse accessions comprise 23 wild soybeans (*G. soja*), 45 landraces, and 25 improved cultivars and represent primary founder lines of U.S. soybean breeding programs^46^. When compared with known salt-tolerant and sensitive-lines from 106 germplasm accessions, we identified 23 lines which were clustered with salt-tolerant accessions and the remainder with salt-sensitive genotypes. These lines were assigned to three structural variant (SV) groups (Supplementary Table S2).

### Identification of structural variants associated with salt-tolerant and sensitive groups

**Figure 4:**
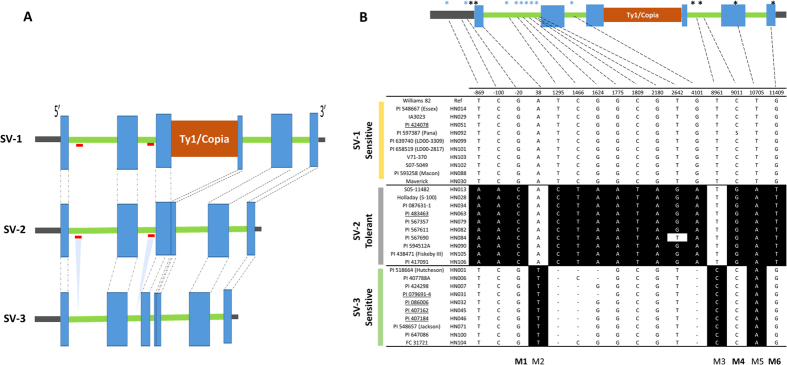
(**A**) Structural variation in the *GmCHX1* gene. Blue box represents exon, green bar represents intron, brown box represents insertion of Ty1/Copia transposon in exon 3, and gray bar represents 3′ and 5′ UTR. Dotted lines indicate the exon position. Red lines indicate that this region is deleted in SV-3 (Valliyodan *et al.* unpublished). (**B**) Schematic graph shows the position of SNP/Indels at for the *GmCHX1* (40621077-40634451) gene. For clear visualization, 10 genotypes from each SV group were selected (for all other lines see Supplementary Table S3). SNP in back background are different from the reference genome (W82). The asterisk (*) above gene structure represents the approximate position of the SNP. SNPs used for the KASPar assay is denoted by black asterisk. The number above the SNP matrix shows position from start codon. PI line with underline represents wild soybean genotypes (*G. soja*).

*GmCHX1* belongs to the sodium/hydrogen (Na^+^/H^+^) exchanger family and comprises 10 transmembrane domains (TMD) (Supplementary Fig. S5). Recently, an improved assembly of the soybean genome was released (http://phytozome.jgi.doe.gov/pz/portal.html), and the gene *Glyma03g32900* was predicted to produce two transcript models (*Glyma.03G171600*, *Glyma.03g171700*) in the new soybean genome assembly (Wm82.a2.v1). However, in the first assembly (W82.a1.v1), this gene was predicted to have a single transcript. This prediction of two transcripts could be due to considering an alternative spliced model as two separate genes. In this study, we used assembly one for consensus alignment and further genotypic inference. To infer allelic variation, the ~13 Kb consensus sequence of the gene *GmCHX1*, including the gene plus a 2 Kb upstream promoter region, was aligned with the soybean reference genome. Based on 100% similarity, the alignment with the reference genome revealed three major structural variants (SV) - SV-1, SV-2 and SV-3 (Fig. 4A) and several SNPs (Fig. 4B, Supplementary Table S3). SV-1 was similar to the salt-sensitive W82, C08^25^, as well as 85–140 genotypes^24^ that retain ~3.3 Kb Ty1/copia retrotransposon in exon 3. The presence of the Ty1/copia retrotransposon results in loss-of-function, leading to a salt-sensitive genotype. In contrast, SV-2 does not carry the Ty1/copia retrotransposon and hence expresses full-length protein (811 aa residue, NCBI ID KF879911.1), confirming the salt-tolerant genotype^25^. SV-1 and SV-2 have been reported in earlier studies^24^,^25^, whereas SV-3 was identified in this study. Genotypes belonging to SV-3 also lack the Ty1/copia retrotransposon; however, phenotypically, this group of lines is sensitive to salt treatment. To understand the phenotypic differences between the SV-2 and SV-3 lines, we studied the variation in the promoter and coding regions of these two groups. It is known that SNPs in the coding or promoter regions can abolish protein localization and function. We identified 29 SNPs in the promoter region and nine non-synonymous SNPs leading to an amino acid change specific to the SV-3 group (Supplementary Fig. S3). Three out of nine nonsynonymous SNPs (at amino acid position 13, 354 and 450) were identified at a high frequency in the SV-3 group compared with the other six SNPs (Supplementary Fig. S3). Furthermore, an ~180 bp deletion (∆232–292 aa) in exon 3 and two large deletions in the first and second introns were identified (Fig. 4A, indicated by a red line) that were confined to SV-3, with exception of genotype HN058 (PI 438258). Based on a transmembrane topology prediction tool, *GmCHX1* comprises 10 transmembrane domains (TMD), and the deletion starting at 232 aa resulted in the loss of the seventh transmembrane helix domain (Supplementary Fig. S5). Qi *et al.*^25^ also identified a deletion in exon 3, but this deletion was smaller than those that we observed, possibly due to the different sets of soybean lines used in each both study. Haplotype analysis using SNP information from the 129 lines (106 re-sequenced lines and 23 out of 31 lines from Lam *et al.*^44^ with known salinity reactions) was performed (Supplementary Fig. S4). Overall, allelic variation other than the Ty1/copia retrotransposon insertion could be the reason for salt-sensitive genotypes in SV-1, although further studies are required to confirm this inference.

### Discovery of informative SNP markers associated with the salt-tolerance gene

The WGRS and phenotypic correlation information of 104 soybean lines were used to identify SNP marker(s) associated with the salt-tolerance locus. First, SNPs associated with three allelic variants (Fig. 4B) were considered, including three SNPs located in the promoter region (the 5′ UTR (untranslated region)) and, thirteen in the genic region (introns and exons). To evaluate which SNP correlated with the three structural variants, six SNPs - M1 (−20 bp, promoter), M2 (38 bp, first exon), M3 (8961 bp, third intron), M4 (9011 bp, third intron), M5 (10705 bp, fifth exon) and M6 (6^th^ exon) - were selected for KASPar assay design. Based on genotypic data, M2 and M3 were able to differentiate between SV-1, SV-2 with SV-3 suggesting that these markers were associated with ‘Hutcheson type’ (i.e. SV-3, without transposon insertion) salt-sensitive genotypes. The M5 SNP was associated with Ty1/copia retrotransposon insertion and differentiated between SV-1 vs. SV-2 and SV-3. Importantly, the SNP markers M1, M4 and M6 were associated with salt-sensitive (SV-1 and SV-3) and salt-tolerant (SV-2) lines.

### Validation of makers in diverse germplasm and interspecific population

**Figure 5:**
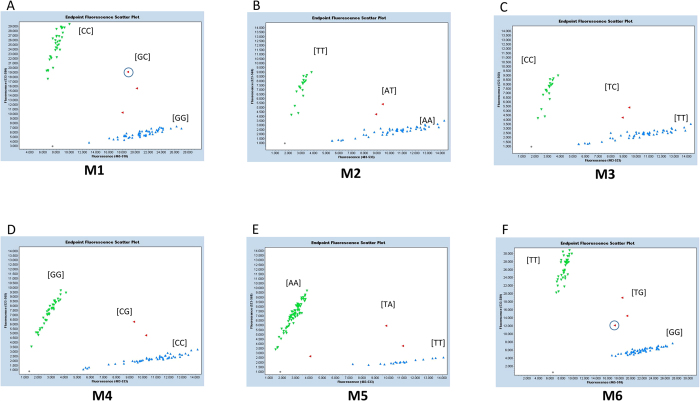
Sample genotyping plots of a diverse set of 106 WGRS lines and F_8_ RILs from population (PI 483463 x Hutcheson). Plots generated from Roche 480 II software during KASPar assay genotyping of M1-M6 SNP markers. A-E: KASPar SNP graphs of 106 diverse soybean lines. F: KASPar SNP graph of RIL population. Genotype signal: Green- Mutant, Red- Heterozygote, Blue- WT (W82), Grey- Non template control. Heterozygote signal highlighted by circle represents artificial heterozygote.

Three KASPar assays (M2, M3 and M5) were selected to identify the structural variants representative of three groups. The M2 and M3 marker assays precisely differentiated SV-3 from SV-1 and SV-2 with a >98% success rate (Supplementary Table S4; Fig. 5B,C). Similarly, the marker M5 differentiated between a transposon insertion allele (SV-1) and a non-insertion allele (SV-2, -3) with a >98% success rate (Fig. 5E). The SNP genotypes were found to be in complete agreement with the three structural variant groups (Supplementary Table S3). To evaluate the genotype-phenotype correlation for salt tolerance in diverse lines, the salt tolerance phenotypic data of 104 lines were tested with three (M1, M4 and M6) KASPar assays (Fig. 5A,D). A strong correlation was observed between SNP genotype and reaction to salt, with the exception of 10 lines (success rate >91%). However, the success rates of genotyping for high tolerance (cluster 1) and sensitivity (cluster 4) were 95 and 100%, respectively (Fig. 1C, Supplementary Tables S1 and S4). In agreement with the previous studies^24^,^47^, Peking (HN002) carries salt-tolerant alleles similar to those of other salt-tolerant lines; however, phenotypically, this line was found to be salt-sensitive (SPAD ratio 0.67, LSS 3.6). On the basis of hierarchical clustering, Peking grouped with the S-100 line (SV-2), suggesting that this gene might be suppressed after transcription or that its expression might be regulated by unknown *cis* or *trans*-elements; however, further study is needed to validate these observations.

In addition to 104 diverse sequencing lines, we performed a precise genotyping test on an interspecific bi-parental population of F_8_ recombinant inbred lines (RILs) from a PI 483463 x Hutcheson cross^48^,^49^ (Fig. 5F; Supplementary Table S5). The parental line PI 483463 (HN063) is a wild soybean accession (*G. soja*) and carries a salt tolerance allele (SV-2), while Hutcheson (HN001) carries a salt sensitivity allele (SV-3) (Fig. 3). HN063 (PI 483463) and HN028/IGDB-129 (S-100) are considered highly salt-tolerant lines with the common ancestor S-100, according to the U.S. breeding programs^15^,^48^. Plant reactions to salt treatment showed that Hutcheson exhibited severe leaf scorch; however, the leaves of PI 483463 were less affected by salt injury (Fig. 1; Supplementary Table S1). We also tested M1 and M6 markers on artificial heterozygous DNA, in which the DNA of tolerant (HN105 or HN063) and sensitive (HN001 or Williams 82) accessions were mixed at equal 10 ng concentrations. This artificial heterozygote allele correctly designated the genotype and was clustered between mutant and wild-type alleles (Fig. 5A,F). Overall, a strong association was observed between SNP genotype and reaction to salt treatment in the RIL population at a success rate >94.5%. This shows that the gene-based molecular markers and the genotyping assay developed in this study are powerful and efficient tools for selecting true heterozygotes in an early generation (F_2_) for genetic studies or breeding purposes, as well as for selecting tolerant genotypes from diverse soybean germplasm.

## Discussion

An important goal of whole genome re-sequencing data analysis of crop species is to explore genetic variation in diverse germplasm resources, such as wild species, landraces and improved cultivars and to identify molecular markers that accelerate breeding cycles. Soybean germplasm, both *G. max* and *G. soja* species, provide a wide range of salt tolerances. For many years, a great effort has been made to understand the mechanism of salt reaction^39^ and to precisely identify gene(s) underlying salt tolerance QTL in soybeans^24^,^25^. Previous studies have shown that a QTL on Chr. 3 is the major genomic region determining salinity tolerance in soybean. This locus carries the dominant functional sodium/hydrogen exchanger family gene *Glyma03g32900* (*GmCHX1*) and accounts for more than 64% of the phenotypic variation^25^. GWAS is a statistically powerful approach and provides a higher resolution to identify the position of genetic factors underlying the trait of interest^45^,^50^,^51^,^52^. A large number of GWAS has been successfully conducted in soybean using SoySNP50K and WGRS data for nematode resistance^50^, carbon-isotope^51^, oil and protein content^45^,^52^, and domestication traits^45^. In this study, GWAS pin-pointed a single major and significant locus on Chr. 3 that harbors the previously characterized *GmCHX1* gene. While the SoySNP50K and WGRS data were able to identify the same major loci on Chr. 3, the number of SNPs was relatively higher in WGRS dataset, which was not surprising. Importantly, high quality WGRS data benefited the discovery of novel structural variants and the large number of SNPs that were translated into functional markers. The results obtained from GWAS thus, allowed us to further investigate the haplotype and SNP variation using WGRS datasets. The genetic basis of salt tolerance in soybean is relatively less complex compared with the response to other abiotic stresses (e.g., drought, flooding^1^,^53^) due to the presence of a single dominant locus as detected in the present study.

In addition to genotypic data, robust salt tolerance assays for generating reliable phenotypic data are also important for molecular marker development. A conventional method of screening for salt tolerance in soybean was based on visual leaf scorch score (LSS). However, in the present study, we utilized a combination of LSS and leaf chlorophyll assessed by a SPAD ratio to determine the phenotypes. The chlorophyll content of a developing plant changes significantly under stress conditions, and chlorophyll level has been shown to be a good indicator of photosynthetic function^10^,^12^. Chlorophyll fluorescence provides a non-invasive and rapid method for estimating the photosynthetic performance of plants^54^,^55^. Lenis *et al.*^8^ reported that incremental increases in NaCl concentration from 25 to 100 mM were significantly associated with leaf scorch and SAPD ratio. In this study, the significant negative correlation between LSS and SPAD ratio gave us additional confidence in associating the phenotype with the genotype.

The function of *GmCHX1* was studied by expressing a tolerant allele using transgenic soybean hairy roots and transgenic tobacco BY-2 cells^25^. This analysis revealed healthy hairy roots and a higher survival rate for BY-2 cells in the transgenic lines, which confirmed a gain-of-function. The *GmCHX1* gene is expressed under elevated salt conditions in root stellar cells and limits salt transport to shoot tissues^24^. In other plant species such as cotton^56^, rice^57^, Arabidopsis^58^,^59^, *P. tenuiflora*^60^, wheat^17^,^61^ and grapevine^62^, the expression of Na^+^ exclusion protein in root tissue is associated with lower Na^+^ accumulation in shoot tissue. In general, a plant adapts to soil salinity through osmotic tolerance, Na^+^ or Cl^−^ exclusion, and the accumulation of ions in various tissues^10^,^12^,^63^,^64^. Consistent with earlier findings^8^, significantly lower Na^+^ accumulation in the leaf tissues of tolerant genotypes was observed (Supplementary Fig. S1), confirming sodium exclusion in the above-ground tissues of soybean plants. The tolerant genotypes had a lower LSS, greater SPAD ratios and a greater capacity to prevent Na^+^ transport from the soil to stems and leaves than did sensitive lines.

Wild relatives represents a unique resource to study the regulation of salt tolerance and other abiotic stress responses and present a wide range of genetic diversity for several traits^8^,^16^,^65^,^66^. The progeny of a cross between *G. max* and *G. soja* were more tolerant to salt injury than those of a cross between *G. max* and *G. max*^48^, suggesting allelic and background effects. Previously, Lee *et al.*^48^ carried out an allelism test and concluded that wild soybean has a tolerant locus different from that in the line S-100 (HN028)^48^,^49^. However, they subsequently mapped this trait to a similar genomic region (Chr. 3) and concluded that the tolerance gene from the two sources could be the same, but the degree of tolerance (after 30 days of salt tolerance) was different^21^. In agreement with previous studies, we confirmed that wild and cultivated soybean possess the same loci but show allelic variation (Fig. 4). Therefore, differential responses of salt-tolerant genotypes could be (1) the result of allelic variation in promoter and gene regions^25^; (2) due to mechanisms used to exclude sodium ions from the roots, thereby preventing accumulation at toxic concentrations in the stem and leaves^10^,^12^,^62^; (3) caused by regulation at the transcription or post-transcriptional levels^13^; or (4) due to genetic background effects^8^. Qi *et al.*^25^ concluded that elimination of the salt tolerance gene in salt-sensitive germplasm could be due to negative selection against a stress tolerance gene in an unstressed environment because its expression could be an energy burden on the plant when its function is not required.

Guan *et al.*^24^ identified nine haplotypes, including two salt-tolerant haplotypes and seven salt-sensitive haplotypes, based on SNP variation in *GmSALT3* (*GmCHX1*) and its ~600 bp promoter region. In this study, we utilized high-quality, deep sequence information (15X) for *GmCHX1* loci (gene plus 2 kb up- and down-stream sequence) and identified three major structural variants and several SNPs (Supplementary Table S3). A number of SNPs identified in this study matched with previously reported SNPs. However, two insertions of 148 bp and 4 bp in the promoter region reported by Guan *et al.*^24^ were not observed in our sequenced lines, in agreement with a re-sequencing analysis by Qi *et al.*^25^.

In previous studies^15^,^22^,^47^, SSR and SCAR (Sequence Characterized Amplified Region) markers were utilized in association with salinity tolerance based on the sequence information obtained by mapping parents to genotype diverse germplasm. Several SSR marker alleles were found to be associated with salt tolerance phenotypes in the descendants and diverse germplasm. A majority of SSRs can amplify multiple alleles at one locus depending on the genetic background, despite the fact that the same allele may not always be associated with that particular trait^47^,^67^. In addition, popular PCR or non-PCR based markers, including SSR and SCAR makers, have limitations for use in high-throughput genotyping, such as high cost and transferability in complex genomes and diverse germplasm. In next-generation breeding, these markers cannot be used in high-throughput genotyping technologies as required by breeders to accelerate selective breeding for a number of traits. Correct identification and quality assurance are crucial to ensure reproducible breeding programs. Thus, next-generation SNPs along with the KASPar genotyping method offer a wide range of advantages over other molecular markers^68^. KASPar assays have emerged as a powerful tool due to their high-throughput nature, locus specificity, co-dominant inheritance, simple documentation, transferability between genotyping platforms, lower error rate and lower cost^30^,^36^. Moreover, KASPar assay can be applied to germplasm characterization, allele mining, and fore-ground and back-ground selection^34^.

Remarkably, the genotypic and phenotypic data generated in the present study are more resilient and provided a solid foundation to develop robust, high-throughput, and breeder-friendly markers. We successfully identified and validated several SNP-based KASPar assays for salt-tolerance using WGRS information with a >95% prediction rate (Supplementary Table S1). The KASPar assay was developed to identify not only the salt-tolerant and sensitive genotypes, but also other structural variants at a high frequency (Supplementary Tables S4 and S5). All KASPar assays were tested on artificial heterozygote DNA and showed a perfect cluster with true heterozygotes (Fig. 5A,F). A few lines (10 out of 106 diverse lines), including the salt-sensitive cultivar Peking (LSS 3.6, SPAD 0.67), did not show an exact correlation between the expected phenotype and the salt tolerance alleles. Several factors may result in this discrepancy between the genotyping and phenotyping results. One reason could be that salinity scoring was based on a 1 to 5 scale, making the aforementioned lines with a moderately-tolerant or moderately-sensitive phenotype difficult to assess. Another possibility is the variation in expression level of the salt-tolerance gene due to unknown (*cis* or *trans*) regulation. In rice, a class of endogenous small RNAs is thought to regulate the expression of salt-responsive genes at the post-transcriptional level^69^. Recently, He *et al.*^10^ elucidated the role of cyclic electron flow into vacuoles under salt stress in soybean and, suggested Na^+^ ion compartmentation mechanism. They identified genes associated with Na^+^ that were highly expressed in the salt-tolerant variety (S111-9) and accumulated Na^+^ in vacuoles, whereas the salt-sensitive variety (Melrose) accumulated Na^+^ in the chloroplasts. In agreement with earlier reports^10^,^24^,^70^, we conclude that in addition to a major salt-tolerant gene (*GmCHX1*), there could be minor undetermined element(s) (e.g., post-transcription regulation or ion compartmentation) involved in salt tolerance in soybean. Future investigation is warranted to understand and elucidate these factors.

**Figure 6:**
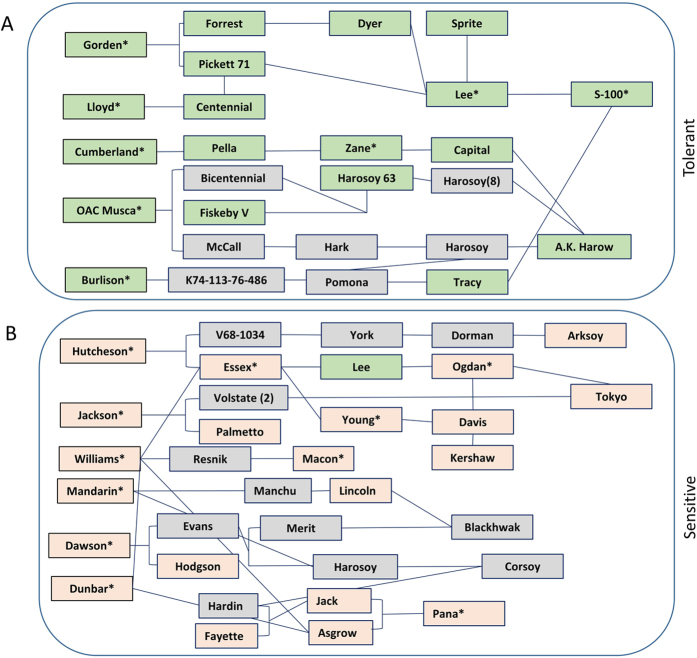
Partial pedigree tracing of salt-tolerant and salt-sensitive lines. Green represents salt-tolerant, light-red represents salt-sensitive, grey unknown. Asterisk (*) denotes that genotype of these lines studied in the present investigation using WGRS information.

Lee *et al.*^15^ reported that several ancestors of U.S. soybean cultivars are salt tolerant; however, our analysis of 93 ancestral lines showed that only 23 lines exhibited a salt-tolerant genotype. The 23 lines included seven wild accessions, nine landraces and seven improved cultivars, including, Gordon (IGDB-228), Lloyd (IGDB-259), Sprite (IGDB-257), Zane (IGDB-234), Capital (IGDB-143), Musca (IGDB-231), and Burlison (IGDB-258). To gain insight into the pedigree information of these lines, we used the GRIN (http://www.ars-grin.gov/npgs/acc/acc_queries.html) and Soybase (http://www.soybase.org/) databases. Interestingly, the pedigree of Gordon, Lloyd, Sprite and Burlison trace back to Lee and S-100 (Fig. 6). The descendants Musca, Zane and Capital can be traced back to the salt-tolerant line A. K. Harrow (Dr. Thomas Carter personal communication). Similarly, the salt-sensitive genotypes trace back to Williams82, Tokyo, Davis and Arksoy (Fig. 6, Supplementary Table S2). This analysis showed that a majority of the U.S. soybean cultivars are fixed for the salt-sensitive allele. To improve salt tolerance, two main approaches can be utilized. The first approach includes the exploration of natural genetic variation via direct selection under saline conditions, either in field or under controlled conditions, or through marker-assisted selection. The second approach includes the generation of transgenic plants expressing a salt-tolerance gene^3^,^71^,^72^. A salt-tolerant transgene (e.g., *GmCHX1*) can also be utilized for positive selection with 150–200 mM NaCl as the selectable agent^73^,^74^. Positive selection offers several advantages over herbicide or antibiotic gene based selection approaches and can be coupled with other transgenes^75^. However, this transgenic approach has several challenges, including acceptance of transgenic crops and the costs associated with regulation and licensing, while screening through marker assisted selection offers several advantages.

In summary, we successfully developed an efficient, high-throughput and cost effective next-generation KASPar assay for salinity tolerance in soybean using a whole genome resequencing information of 106 diverse germplasm lines. The newly developed markers and genotype information will greatly benefit soybean breeders in the development of salt-tolerant varieties. In addition, it will help to select parent lines to design future crosses, trait introgression and the evaluation of diverse germplasm.

## Methods

### Plant materials

A subset of 104 soybean lines were evaluated for salt tolerance in two independent tests in the salinity phenotyping facility at University of Missouri, Columbia, MO, following a well-established procedure as previously described^38^. Briefly, at the V2 growth stage, soybean seedlings in cone-trainers were exposed to salt water (120 mM) added to a depth of one-third (7 cm) of the cone to allow the uptake of salt water. Electrical conductivity (EC) was monitored daily. Individual soybean plants were visually rated for salt sensitivity or tolerance when the sensitive parent, cultivar Hutcheson (HN001), showed severe leaf scorch (approximately 2 weeks after treatment with salt water). Leaf scorch was scored using a 1–5 scale, where 1 = no apparent chlorosis; 2 = slight (25% of the leaves showed chlorosis); 3 = moderate (50% of the leaves showed chlorosis and some necrosis); 4 = severe chlorosis (75% of the leaves showed chlorosis and severe necrosis); and 5 = dead (leaves showed severe necrosis and were withered). The average leaf scorch score for each genotype was calculated using the equation (1):


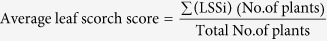


where LSSi = the level of leaf scorch score.

The measurements of leaf chlorophyll content were carried out on the top secondary fully expanded leaves. At 1 day before and 14 d after treatment, the chlorophyll concentration, expressed as SPAD value, was measured with a chlorophyll meter (Konica Minolta SPAD-502). The SPAD ratio, an indicator of the efficiency of the photosynthetic apparatus and shows decreasing chlorophyll content under salt stress, was calculated with a portable fluorometer (model FMS-2 Hansatech Instruments Ltd., England). After scoring LSS and SPAD, leaves, including petiole, were harvested separately before and after salt water treatment. The sodium (Na^+^) content of soybean leaves for the five genotypes with known levels of salt tolerance was measured in two independent experiments as described by Lenis *et al.*^8^ using a Perkin-Elmer (Wellesley, MA, USA) atomic absorption spectrophotometer^76^.

In addition to the subset of 104 germplasm lines, the salt phenotypic data of an F_8_ RIL population developed from an interspecific cross of PI 483463 and Hutcheson was obtained from a previous study^48^. These phenotypic data were employed to test the association of the phenotypes and genotypes that were generated in the present study.

### Genome-wide association study

The WGRS data of 106 lines (~9.4 million SNPs) and SoySNP50K iSelect BeadChip data^42^ was utilized for GWAS analysis. The WGRS data (sequencing depth ~15X) for 106 lines was obtained from Soybean Genetics and Genomics Laboratory at the University of Missouri (Valliyodan *et. al.* unpublished), and the SoySNP50K data was accessed from the soybean database (http://www.soybase.org/). After excluding SNPs with more than 10% missing data and a minor allele frequency (MAF) less than 5%, over 5 million SNPs from WGRS and 37,400 SNPs from SoySNP50K data were used for GWAS. The PCA matrix and identity by descent (IBD) were calculated from LD-pruned SNPs in SVS v8.1.5 (http://goldenhelix.com/SNP_Variation/). A single-locus mixed linear model developed by the EMMAX method and implemented in SVS v8.1.5 was used. The EMMAX model corrects for population structure as well as identity by descent (IBD)^41^. We used a PCA matrix (first two vectors) and the IBD matrix to correct for population stratification. We defined the whole-genome significance cutoff as empirical^77^ threshold 7.3 (*p* = 5 × 10^−8^) for selection of significant markers. Manhattan plots for associated SNPs were visualized in GenomeBrowse v1.0 (Golden Helix, Inc).

### Analysis of structural variation

The mapped sequence reads of 106 lines at position (Chr. 3:40621077-40634451) were used to create a consensus sequence using SAM and BAM tools^78^. The consensus sequence was then aligned with the soybean reference genome W82 (Phytozome: Gmax v9.0) using the MEGA 6.0^79^ and BioEdit^80^ sequence alignment editor tools. Transcript sequence-based annotation^25^ was used to identify structural variants (SV-1, SV-2 and SV-3) associated with the *GmCHX1* gene. SNPs were identified using an in-house SOAP3^81^ pipeline and were confirmed by examining read alignment in the GenomeBrowse tool (http://goldenhelix.com/GenomeBrowse/). SNPs were further analyzed for possible synonymous/non-synonymous variation by translation into amino acid sequences.

The publically available WGRS datasets of 31 lines^44^ and 93 US ancestral lines^45^ were downloaded to investigate genetic variation. SNP haplotypes were examined by generating map and genotype data files using TASSEL 5.0 program^82^ and clustering pictorial output for *GmCHX1* genic region was visualized using FLAPJACK and SNPviz software^83^,^84^.

### SNP and KASPar assay design

Whole-genome re-sequencing coupled with structural variation information were used to develop KASPar assays. A panel of six SNPs (Fig. 4B) were selected and targeted for the development of the KASPar assays. Two allele-specific forward primers with tail sequences and one common reverse primer were synthesized for the SNP genotyping assays (Supplementary Table S6). The reaction mixture was prepared according to the protocol described by KBiosciences (http://www.ksre.ksu.edu/igenomics). Briefly, KASPar assays were run in a 10 μl final reaction volume containing 5 μl KASPar master mix, 0.14 μl primer mix, 2 μl 10–20 ng/μl genomic DNA, and 2.86 μl water. The following cycling conditions were used: 15 min at 95 °C, followed by 10 touchdown cycles of 20 s at 94 °C, 1 min at 61–55 °C (dropping 0.6 °C per cycle); and then 26 cycles of 20 s at 94 °C, 1 min at 55 °C. The fluorescent end-point genotyping method was carried out using a Roche LightCycler 480-II instrument (Roche Applied Sciences, Indianapolis, IN, USA). The seeds of 104 diverse lines and a RIL population derived from a PI 483463 x Hutcheson cross were germinated in a greenhouse with 24 seeds per line. Young leaf tissue from each line was pooled and flash-frozen in liquid nitrogen. DNA was isolated using a modified C-TAB extraction protocol.

### Statistical analysis

Comparisons between the mean treatment values were made by least significance difference (LSD) using Duncan’s multiple test.

## Additional Information

**How to cite this article**: Patil, G. *et al.* Genomic-assisted haplotype analysis and the development of high-throughput SNP markers for salinity tolerance in soybean. *Sci. Rep.* **6**, 19199; doi: 10.1038/srep19199 (2016).

## Supplementary Material

Supplementary Information

## Figures and Tables

**Table 1:** Relationship of four phenotype clusters with an average leaf chlorophyll content (SPAD ratio) and leaf scorch score (LSS).

Cluster	Salt reaction	No. ofgenotypes	SPADratio	LSS
I	Tolerant	21	1.01^a^	1.21^d^
II	Moderately tolerant	14	0.91^b^	2.34^c^
III	Moderately sensitive	49	0.70^c^	3.66^b^
IV	Sensitive	20	0.55^d^	4.72^a^
CV%			19.48	14.24

Mean followed by the same letter are not significantly different according to Duncan’s multiple range test (*p* < *0.01*). CV = coefficient of variation.
