# Supplementary material for: Genomic-assisted haplotype analysis and the development of high-throughput SNP markers for salinity tolerance in soybean
Source: Sci Rep. 2016 Jan 19;6:19199. doi: 10.1038/srep19199 (PMC4726057; doi:10.1038/srep19199)
Supplement: Supplementary Information [file srep19199-s1.pdf]

Genomic-assisted haplotype analysis and development of high-throughput SNP markers for salinity tolerance in soybean

Gunvant Patil[1,#], Tuyen Do[1,#], Tri D. Vuong[1], Babu Valliyodan[1], Jeong-Dong Lee[2], Juhi Chaudhary[1], J. Grover Shannon[1], Henry T. Nguyen[1,*]

Supplementary Table S1: (A) Phenotypic evaluation of chlorophyll concentration (SPAD ratio) and leaf scorch score (LSS) of 104 resequencing lines for salinity injury using cone-tainer method. Phenotype clusters were correlated with allelic variants. **(B)** List of top five SNPs significantly identified in SoySNP50K data, associated with LSS and SPAD based on EMMAX ($-\log_{10} P \geq 7.3$) and r^2 values.

(A)

			Phenotype					Genotype			
**Seq Id***	**Accession***	**Plant Id**	**SPAD - B^**	**SPAD - A^**	**SAPD Ratio^**	**LSS**	**Phenotype Cluster#**	**M1**	**M4**	**M6**	**Genotype vs Phenotype**
HN009	PI 495017C	Beijing da qing don	35.75	35.64	1.00	1.0	1	C	G	T	Matched
HN013	PI 661090	S05-11482	37.96	38.10	1.00	1.1	1	C	G	T	Matched
HN015	PI 437654	Er-HNej-jA-	35.42	39.37	1.11	1.3	1	C	G	T	Matched
HN021	PI 209332	No. 4	32.06	34.46	1.08	1.3	1	C	G	T	Matched
HN024	PI 548316	Cloud	34.96	38.09	1.09	1.3	1	C	G	T	Matched
HN027		S10-11227	34.84	34.27	0.98	1.3	1	C	G	T	Matched
**HN028**	PI 572239	Holladay (S-100)	37.26	33.54	0.90	1.4	1	C	G	T	Matched
HN039	PI 366121		33.35	33.23	1.00	1.4	1	G	C	G	Not Matched
HN055	PI 437679	Nan-cou	30.54	30.10	0.99	1.5	1	C	G	T	Matched
HN056	PI 437863A	DV-2841	32.84	33.40	1.02	1.4	1	C	G	T	Matched
HN058	PI 458515	Tie Zhugan	34.43	33.11	0.96	1.0	1	C	**G**	T	Matched
**HN063**	PI 483463		35.04	32.79	0.94	1.0	1	C	G	T	Matched
HN067	PI 548317	Columbia (T38)	34.46	37.60	1.09	1.0	1	C	G	T	Matched
HN069	PI 548415	Sooty (PI 16790B)	32.71	34.75	1.06	1.3	1	C	G	T	Matched
HN074	PI 561271	Pei xian da quing dou	35.38	35.86	1.01	1.1	1	C	G	T	Matched
HN075	PI 567230	WJK-PRC-23	28.96	33.10	1.14	1.3	1	C	G	T	Matched
HN079	PI 567357	Du jia qiao huang dou	34.30	32.25	0.94	1.0	1	C	G	T	Matched
HN080	PI 567383	Da ke huang dou	34.22	33.68	0.98	1.2	1	C	G	T	Matched
HN085	PI 567719	Fu yang (43)	38.64	37.19	0.96	1.0	1	C	G	T	Matched
HN090	PI 594512A	Bian zi jiang se dou	35.66	35.67	1.00	1.2	1	C	G	T	Matched
**HN105**	PI 438471	Fiskeby III	38.65	35.45	0.92	1.2	1	C	G	T	Matched
HN008	PI 437655	Er-huan-jan	33.11	27.57	0.83	2.7	2	C	G	T	Matched
HN010	PI 468915		27.99	33.56	1.20	2.0	2	C	G	T	Matched
HN019	PI 548402B		38.86	35.24	0.91	2.0	2	C	G	T	Matched
HN025	PI 567516C	Ba yue zha	29.19	29.05	1.00	2.7	2	C	G	T	Matched
HN040	PI 378702		35.35	27.35	0.77	2.1	2	G	G	G	Matched

HN047	PI 407729		33.13	30.60	0.92	2.4	2	C	G	T	Matched
HN060	PI 467312	Cha-mo-shi-dou	34.79	30.75	0.88	2.8	2	C	G	T	Matched
HN076	PI 567336B	Lao hei dou	28.83	26.97	0.94	2.3	2	C	G	T	Matched
HN077	PI 567343	Ma huang dou	30.76	31.23	1.02	1.7	2	C	G	T	Matched
HN082	PI 567611	Ba yue zha	35.00	34.82	0.99	2.4	2	C	G	T	Matched
HN084	PI 567690	Fu yang (7)	36.74	33.31	0.91	2.0	2	C	G	T	Matched
HN086	PI 567731	Fu yang (56)	43.20	33.50	0.78	2.5	2	C	G	T	Matched
HN088	PI 593258	Macon	37.63	27.13	0.72	2.6	2	G	C	G	Not Matched
HN101	PI 658519	LD00-2817	31.27	27.73	0.89	2.6	2	G	C	G	Not Matched
HN002	PI 548402	Peking	34.56	23.04	0.67	3.6	3	C	G	T	Not Matched
HN003	PI 089772	7193	35.04	25.23	0.72	3.7	3	G	C	G	Matched
HN004	PI 090763	7570	33.53	21.93	0.65	4.1	3	G	C	G	Matched
HN005	PI 404166	Krasnoarmejskaja	35.53	26.84	0.76	3.1	3	G	C	G	Matched
HN006	PI 407788A	SG1P	30.37	17.14	0.56	3.7	3	G	C	G	Matched
HN007	PI 424298	KAS 300-10	27.27	19.85	0.73	3.5	3	G	C	G	Matched
HN014	PI 548667	Essex	42.10	29.20	0.69	2.9	3	G	C	G	Matched
HN016	PI 567387	Huang huai dou	34.11	29.52	0.87	3.1	3	C	G	T	Not Matched
HN017	PI 437725	Te-zu-gan	35.84	30.85	0.86	3.2	3	C	G	T	Not Matched
HN018	PI 437690	Pin-din-guan	29.36	27.47	0.94	3.1	3	C	G	T	Not Matched
HN020	PI 088788	5913	32.73	28.53	0.87	3.0	3	C	G	T	Not Matched
HN022	PI 404198B	Sun huan do	30.13	29.01	0.96	3.1	3	C	G	T	Not Matched
HN023	PI 424608A	Sun huan do	32.03	27.11	0.85	3.9	3	G	C	G	Matched
HN030	PI 639740	Maverick	37.23	20.16	0.54	4.1	3	G	C	G	Matched
HN031	PI 079691-4	No name	24.81	20.11	0.81	4.0	3	G	C	G	Matched
HN032	PI 086006	Kiio Shokuzu	30.39	21.42	0.70	3.9	3	G	C	G	Matched
HN035	PI 196175	Yu tae	35.54	18.52	0.52	4.0	3	G	C	G	Matched
HN036	PI 200471	Hanayome Ibaragi No. 1	38.68	25.53	0.66	4.1	3	G	C	G	Matched
HN037	PI 200508	Natsu Daizu	35.23	22.21	0.63	4.3	3	G	C	G	Matched
HN038	PI 248515		35.23	21.19	0.60	3.4	3	G	C	G	Matched
HN041	PI 398593	KAS 390-4	31.83	23.89	0.75	4.0	3	G	C	G	Matched
HN042	PI 398595	KAS 390-5	36.47	21.88	0.60	3.8	3	G	C	G	Matched
HN043	PI 398610	KAS 390-8	37.13	23.11	0.62	4.0	3	G	C	G	Matched
HN044	PI 398614	KAS 390-9	35.71	18.69	0.52	4.0	3	G	C	G	Matched
HN046	PI 407184	K29	36.37	26.30	0.72	3.0	3	G	C	G	Matched
HN050	PI 416937	Houjaku Kuwazu	34.23	25.33	0.74	3.6	3	G	C	G	Matched
HN052	PI 424079		34.71	24.43	0.70	3.9	3	G	C	G	Matched

HN054	PI 437169B	VNIISC-4	31.63	22.03	0.70	4.0	3	G	C	G	Matched
HN061	PI 471938	0197	38.54	26.07	0.68	4.0	3	G	C	G	Matched
HN062	PI 475783B	Tsing 2	30.06	21.41	0.71	3.9	3	G	C	G	Matched
HN065	PI 542044	Kunitz (L81-4590)	37.10	23.04	0.62	4.1	3	G	C	G	Matched
HN066	PI 547862	L83-570	39.45	21.83	0.55	4.0	3	G	C	G	Matched
HN070	PI 548511	Beeson 80	36.40	22.15	0.61	3.3	3	G	C	G	Matched
HN072	PI 549031	No name	34.60	24.99	0.72	3.6	3	G	C	G	Matched
HN073	PI 552538	Dunbar	39.24	25.13	0.64	3.4	3	G	C	G	Matched
HN081	PI 567519	Bai hua chi	37.41	21.13	0.56	3.6	3	H	C	H	Matched
HN087	PI 591539	L91-8558	38.09	25.58	0.67	2.8	3	G	C	G	Matched
HN089	PI 594012	Heuksatangdu?	32.05	25.33	0.79	3.7	3	G	C	G	Matched
HN091	PI 594599	Chang de chun hei dou	40.09	23.95	0.60	4.0	3	G	C	G	Matched
HN092	PI 597387	Pana	33.01	25.93	0.79	3.9	3	G	C	G	Matched
HN094	PI 603170	GL 2683 /96	31.41	23.44	0.75	3.6	3	G	C	G	Matched
HN095	PI 603175	GL 2688 /96	26.81	24.87	0.93	3.2	3	G	C	G	Matched
HN096	PI 603176A	No name	35.06	24.52	0.70	3.8	3	G	C	G	Matched
HN098	PI 605869A	Sample 140	36.59	21.32	0.58	4.0	3	G	C	G	Matched
HN099	PI 639740	LD00-3309	37.42	24.89	0.67	3.2	3	G	C	G	Matched
HN100	PI 647086	N8001	38.63	23.49	0.61	3.6	3	G	C	G	Matched
HN102		S07-5049	35.23	19.04	0.54	4.0	3	G	C	G	Matched
HN103	V71-370	No name	33.42	23.33	0.70	4.0	3	G	C	G	Matched
HN106	PI 417091	Kuro mame	30.23	28.78	0.95	3.7	3	C	G	T	Not Matched
**HN001**	PI 518664	Hutcheson	39.26	19.61	0.50	4.6	4	G	C	G	Matched
HN011	PI 507354	Tokei 421	39.77	13.47	0.34	5.0	4	G	C	G	Matched
HN012	PI 567305	Hei dou zi	33.44	18.38	0.55	5.0	4	G	C	G	Matched
HN026	PI 612611	Browngilgun	36.24	19.93	0.55	5.0	4	G	C	G	Matched
HN029		IA3023	39.27	18.40	0.47	4.4	4	G	C	G	Matched
HN033	PI 087617	Miyongaikon	40.03	19.04	0.48	4.8	4	G	C	G	Matched
HN045	PI 407162	K1-D	32.69	19.96	0.61	4.4	4	G	C	G	Matched
HN048	PI 407965	KAERI 504-4	39.14	21.54	0.55	4.6	4	G	C	G	Matched
HN049	PI 408105A	KAS 633-19	39.14	20.19	0.52	4.8	4	G	C	G	Matched
HN051	PI 424078	74077	35.43	17.47	0.49	5.0	4	G	C	G	Matched
HN053	PI 424088		36.17	15.05	0.42	5.0	4	G	C	G	Matched
HN057	PI 438258	VIR 4714	35.84	24.54	0.68	4.4	4	G	C	G	Matched
HN064	PI 518751	NS-20	37.54	26.01	0.69	4.4	4	G	C	G	Matched
HN068	PI 548349	Ilsoy	34.50	19.49	0.56	4.9	4	G	C	G	Matched

HN071	PI 548657	Jackson	40.61	17.04	0.42	5.0	4	G	C	G	Matched
HN078	PI 567354	You huang dou	29.34	19.69	0.67	4.5	4	G	C	G	Matched
HN083	PI 567651	Shang cai er cao ping ding shi	33.15	24.65	0.74	4.5	4	G	C	G	Matched
HN097	PI 603497	Hua dou	29.70	17.73	0.60	4.7	4	G	C	G	Matched
HN104	FC 31721	No name	35.81	18.20	0.51	5.0	4	G	C	G	Matched
HN107	PI 417015	Kawanagare (Iwate)	37.02	22.28	0.60	4.6	4	G	C	G	Matched
Reference	PI 518671	Williams 82	35.84	24.54	0.68	4.30	-	G	C	G	Matched

Note: Highlighted sequence id represents lines with known salinity reaction. Accession with underline represents wild soybean lines.*
"H" represents heterozygote.
# Cluster 1: Tolerant; 2: Moderately tolerant; 3: Moderately sensitive; 4: Sensitive
^ SPAD-A: chlorophyll concentration 1 d before treatment; SAPD-B: chlorophyll concentration 14 d after treatment; SPAD-Ratio: SPAD-A/SPAD-B.

(B)

	Chr: SNP position	Allele		$-log_{10}$ (P-value)	r^2	*MAF*
		Major	Minor			
LSS (WGRS)	3:40632077	G	C	22.62	0.63	0.41
	3:40528845	C	T	21.50	0.61	0.40
	3:40528865	C	T	21.50	0.61	0.40
	3:40493449	C	T	21.22	0.60	0.41
	3:40500559	T	G	21.22	0.60	0.41
SPAD (WGRS)	3:40528845	C	T	17.32	0.53	0.40
	3:40528865	C	T	17.32	0.53	0.40
	3:40483159	C	T	17.07	0.52	0.39
	3:40519933	T	C	17.07	0.52	0.39
	3:40534592	G	A	17.06	0.52	0.39

MAF minor allele frequency.

Supplementary Table S2: Analysis of the *GmCHX1* locus in 93 U. S. ancestral lines[44] using WGRS information [43]. Pedigree information of improved cultivars were assessed using GRIN and Soybase databases. Parental lines with 'red text' represents sensitive and 'green text' represents tolerant genotype.

Seq #	Seq ID	Type	Accession	Geographic region	Variety Name	Cluster*	Pedigree related information
1	IGDB-053	*G. soja*	PI407170	Kyonggi, Korea		SV-3 (S)	
2	IGDB-044	*G. soja*	PI447004	Jilin, China			
3	IGDB-105	*Landrace*	PI594788	Yunnan, China			
4	IGDB-125	*Landrace*	PI567293	Gansu, China			
5	IGDB-118	*Landrace*	PI587552	Jiangsu, China			
6	HN031	*Landrace*	PI079691-4	China			
7	HN006	*Landrace*	PI407788A	South Korea			
8	HN001	*Improved cultivar*	PI518664	USA	Hutcheson		V68-1034 x Essex
9	HN007	*Landrace*	PI424298	South Korea			
10	HN071	*Improved Cultivar*	PI548657	NorthCarolina, USA	Jackson		Volstate(2) x Palmetto, Volstate is from Tokyo x PI54610, Palmetto is from chinese land race with salt susceptble by GRIN. Tokyo is land race from Japan.
11	HN073	*Improved Cutilvar*	PI552538	USA	Dunbar		Platte **X** Asgrow A3127 (Williams X Essex)
12	HN032	*Landrace*	PI086006	Japan			
13	HN045	*G. soja*	PI407162	South Korea			
14	IGDB-109	*Landrace*	PI594579	Hunan, China			
15	IGDB-020	*G. soja*	PI562565	ChollaPuk, Korea			
16	IGDB-116	*Landrace*	PI587752	Hubei, China			
17	IGDB-130	*Landrace*	PI548485	Jiangsu, China			
18	IGDB-108	*Landrace*	PI594615	Guizhou, China			
19	IGDB-107	*Landrace*	PI594629	Guizhou, China			
20	IGDB-229	*Improved cultivar*	PI548985	SouthCarolina, USA	Kershaw		Davis x Hale 3
21	IGDB-094	*Landrace*					
22	IGDB-131	*Improved cultivar*	PI548477	Tennessee, USA	Ogden		Tokyo x PI 54610 PI 54610 is from Changchun, Jilin, China, in 1921
23	IGDB-230	*Improved cultivar*	PI548657	NorthCarolina, USA	Jackson		Volstate(2) x Palmetto, Volstate is from Tokyo x PI54610, Palmetto is from chinese land race with salt susceptble by GRIN. Tokyo is land race from Japan.

24	IGDB-054	*G. soja*	PI407131	Kumamoto, Japan			
25	IGDB-138	*Landrace*	PI548382	Liaoning, China			
26	IGDB-162	*Landrace*	PI407849	ChollaPuk, Korea			
27	IGDB-122	*Landrace*	PI567395	Shaanxi, China			
28	IGDB-166	*Landrace*	PI399043	Cheju, Korea			
29	IGDB-021	*G. soja*	PI562559	ChollaPuk, Korea			
30	IGDB-137	*Landrace*	PI548391	Liaoning, China			
31	IGDB-096	*Landrace*					
32	IGDB-158	*Landrace*	PI423954	Kumamoto, Japan			
33	IGDB-163	*Landrace*	PI407801	Kyonggi, Korea			
34	IGDB-084	*Landrace*					
35	IGDB-262	*Improved cultivar*	PI518664	Virginia, USA	Hutcheson		V68-1034 x Essex
36	IGDB-255	*Improved cultivar*	PI542403	Minnesota, USA	Dawson		Evans x M63-217Y, M63-217Y is a yellow hilum NIL of 'Hodgson'
37	IGDB-167	*Landrace*	PI398296	Kyonggi, Korea			
38	IGDB-126	*Landrace*	PI567258	Jiangxi, China			
39	IGDB-156	*Landrace*	PI424391	ChollaPuk, Korea			
40	IGDB-102	*Landrace*	PI603336	Heilongjiang, China			
41	IGDB-117	*Landrace*	PI587666	Anhui, China			
42	IGDB-106	*Landrace*	PI594777	Yunnan, China			
43	IGDB-097	*Landrace*					
44	IGDB-110	*Landrace*	PI594451	Sichuan, China			
45	IGDB-056	*G. soja*	PI393551	Taiwan			
46	IGDB-114	*Landrace*	PI588053A	Guangdong, China			
47	IGDB-133	*Landrace*	PI548445	Jiangsu, China			
48	IGDB-265	*Improved cultivar*	PI508266	NorthCarolina, USA	Young		Davis/Essex
49	IGDB-035	*G. soja*	PI468916	Liaoning, China			
50	IGDB-048	*G. soja*	PI407288	Jilin, China			
51	IGDB-062	*G. soja*	PI326582A	Primorye, Russian			
52	IGDB-061	*G. soja*	PI339871A	Cheju, Korea			
53	IGDB-037	*G. soja*	PI464935	Jiangsu, China			
54	IGDB-041	*G. soja*	PI458538	Heilongjiang, China			
55	IGDB-047	*G. soja*	PI407301	Zhejiang, China			
56	IGDB-055	*G. soja*	PI407027	Akita, Japan		**SV-1 (S)**	

57	IGDB-050	*G. soja*	PI407275	Kyonggi, Korea			
58	IGDB-042	*G. soja*	PI458536	Heilongjiang, China			
59	IGDB-233	*Improved cultivar*	PI548638	Ontario, Canada	OAC Libra		FH 31-3 (Fiskeby V x Harosoy 63) x Evans Fiskeby V is PI 360955 from Sweden. Harosoy (Mandarin (Ottawa) (2) X A.K. (Harrow))
60	IGDB-236	*Improved cultivar*	PI548604	Missouri, USA	Pershing		D67-3297 [Hill(2) x Kisaya (PI 171450)] x Essex
61	IGDB-242	*Improved cultivar*	PI548524	Iowa, USA	Weber		C1453 [C1266R (Harosoy x C1079) x C1253] x Swift C1079 is from C985 (Lincoln x Ogden), a 'Kent' progenitor C1253 is from Blackhawk x Harosoy
62	IGDB-101	*Landrace*	PI603357	Jilin, China			
63	IGDB-264	*Improved cultivar*	PI513382	Minnesota, USA	Glenwood		Evans x Peterson 85 Peterson 85 is from Provar x (Amsoy x PI 248404) PI 248404 is 'Novosadska Bela' from Yugoslavia in 1958
64	IGDB-193	*Improved cultivar*	FC33243	Iowa, USA	Anderson		A rogue selected from Lincoln by H.J. Anderson, Calamus, Iowa, in 1954
65	IGDB-244	*Improved cultivar*	PI548512	Indiana, USA	Century		Calland x Bonus
66	HN088	*Improved cultivar*	PI593258	NA	Macon		F4 selection from Sherman x Resnik (Asgrow x Williams82)
67	HN099	*Improved cultivar*	PI639740		LD00-3309		'Maverick' x 'Dwight'
68	IGDB-237	*Improved cultivar*	PI548603	Indiana, USA	Perry		Patoka x L37-1355 (a rogue in PI 81041)
69	HN014	*Improved cultivar*	PI548667	USA	Essex		Lee x S5-7075 (N48-1248 x Perry) N48-1248 is from Roanoke x N45-745 (Ogden x CNS)
70	IGDB-135	*Landrace*	PI548406	Jilin, China			
71	HN051	*Landrace*	PI424078	South Korea			
72	HN092	*Improved cultivar*	PI597387	USA	Pana		Jack x Asgrow A3205, Jack (Fayette X Hardin), Fayette (Williams (2) X PI 88788), Hardin (Corsoy (3) X Cutler 71)
73	IGDB-124	*Landrace*	PI567298	Gansu, China			
74	IGDB-140	*Improved cultivar*	PI548362	Illinois, USA	Lincoln		Mandarin x Manchu.
75	HN103	*Improved cultivar*	V71-370	USA			
76	Ref (W82)	*Improved cultivar*	PI518671	USA	Williams 82		Williams(7) x Kingwa
77	IGDB-132	*Landrace*	PI548456	Pyongyang, North Korea			
78	IGDB-139	*Landrace*	PI548379	Heilongjiang, China	Mandarin (Ottawa)		Selected from 'Mandarin' in 1929
79	IGDB-232	*Improved cultivar*	PI548643	Ontario, Canada	Maple Glen		BD22115-13 [(Amsoy x Portage) x Holmberg 840-7-3] x Premier Holmberg 840-7-3 is from 201-14-20 (PI 196491 sib) x 680+993+994 (Muncheberg) Premier is Pride B216 (Corsoy x Wayne)
80	IGDB-256	*Improved cultivar*	PI540552	Ohio, USA	Hoyt		Harcor x Elf
81	IGDB-263	*Improved cultivar*	PI515961	Kentucky, USA	Pennyrile		Williams x Essex

82	IGDB-266	*Improved cultivar*	PI508083	Minnesota, USA	Dassel		Evans x M66-18 M66-18 is from Clay x Altona
83	IGDB-098	*Landrace*					
84	IGDB-103	*Landrace*	PI603318	Heilongjiang, China			
85	IGDB-157	*Landrace*	PI423967	Kumamoto, Japan			
86	HN029	*Improved cultivar*		USA	IA3023		
87	HN030	*Improved cultivar*	PI598124	USA	Maverick		LN86 4668 x Resnik
88	HN101	*Improved cultivar*	PI658519	USA	LD00-2817		Ina x Dwight
89	HN102	*Improved cultivar*		USA	S07-5049		
90	IGDB-015	*G. soja*	PI597459D	Shandong, China			
91	IGDB-060	*G. soja*	PI366120	Akita, Japan			
92	IGDB-031	*G. soja*	PI483464A	Ningxia, China			
93	IGDB-036	*G. soja*	PI468400A	Ningxia, China			
94	IGDB-121	*Landrace*	PI567503	Hebei, China			
95	IGDB-099	*Landrace*					
96	IGDB-022	*G. soja*	PI549046	Shaanxi, China			
97	IGDB-030	*G. soja*	PI483465	Shaanxi, China			
98	IGDB-104	*Landrace*	PI602991	Shandong, China			
99	IGDB-258	*Improved cultivar*	PI533655	Illinois, USA	Burlison		K74-113-76-486 x Century K74-113-76-486 is Tracy x Pomona. Centry from Calland x Bonus.
100	IGDB-231	*Improved cultivar*	PI548644	Ontario, Canada	OAC Musca	**SV-2 (T)**	McCall x Bicentennial (Fiskeby V X Harosoy 63)
101	IGDB-100	*Landrace*					
102	IGDB-120	*Landrace*	PI567525	Shandong, China			
103	IGDB-123	*Landrace*	PI567364	Ningxia, China			
104	IGDB-014	*G. soja*	PI597461A	Shandong, China			
105	IGDB-129	*Landrace*	PI548488	Heilongjiang, China			
106	IGDB-141	*Landrace*	PI548348	Northeast, China			
107	IGDB-143	*Improved cultivar*	PI548311	Ontario, Canada	Capital		No. 171 x A.K. (Harrow) No. 171 is from Sochentze, Heilongjiang, China, in 1931
108	IGDB-144	*Landrace*	PI548298	China			
109	IGDB-228	*Improved cultivar*	PI553047	Georgia, USA	Gordon		Forrest (Dyer X Baggs) **X** Pickett 71, Dyer [Hill X (Lee (2) x Peking)]
110	IGDB-234	*Improved cultivar*	PI548634	Ohio, USA	Zane		Cumberland x Pella
111	IGDB-257	*Improved cultivar*	PI536635	Ohio, USA	Sprite		Williams x Ransom

112	IGDB-259	*Improved cultivar*	PI533602	Arkansas, USA	Lloyd		Selection from Centennial x R75-12L R75-12L is from R72-2647(3) x (D68-18 x PI 88788) R72-2647 is similar to Lee 74 D68-18 is Forrest sib
113	HN084	*Landrace*	PI567690	China			
114	HN105	*Improved cultivar*	PI438471	Sweden	Fiskeby III		
115	HN028		PI572239	USA	Holladay S-100		
116	HN013	*Improved cultivar*		USA	S05-11482		
117	HN082	*Landrace*	PI567611	China			
118	HN063	*G. soja*	PI483463	China			
119	HN106	*Landrace*	PI417091	Japan			

Note: The 93 U.S. ancestral lines ("IGDB") were assigned to structural variant groups after comparison with 106 lines ("HN") used in this study.

Supplementary Table S3 Identification of informative SNPs associated with three different structural variants as shown in Fig. 4. SNP position from start codon.

				M1	M2									M3	M4	M5	M6
Seq id	Cluster	-869	-100	-20	38	1295	1466	1624	1775	1809	2180	2642	4101	8961	9011	10705	11409
W82 (Ref)		T	C	G	A	T	C	G	G	C	G	T	G	T	C	T	G
HN051		T	C	G	A	T	C	G	G	C	G	T	G	T	C	T	G
HN011		T	C	G	A	T	C	G	G	C	G	T	G	T	C	T	G
HN014		T	C	G	A	T	C	G	G	C	G	T	G	T	C	T	G
HN089		T	C	G	A	T	C	G	G	C	G	T	G	T	C	T	G
HN029		T	C	G	A	T	C	G	G	C	G	T	G	T	C	T	G
HN049		T	C	G	A	T	C	G	G	C	G	T	G	T	C	T	G
HN042		T	C	G	A	T	C	G	G	C	G	T	G	T	C	T	G
HN051		T	C	G	A	T	C	G	G	C	G	T	G	T	C	T	G
HN062		T	C	G	A	T	C	G	G	C	G	T	G	T	C	T	G
HN066		T	C	G	A	T	C	G	G	C	G	T	G	T	C	T	G
HN087		T	C	G	A	T	C	G	G	C	G	T	G	T	C	T	G
HN092	SV-1 (S)	T	C	G	A	T	C	G	G	C	G	T	H	T	S	T	G
HN099		T	C	G	A	T	C	G	G	C	G	T	G	T	C	T	G
HN081		T	H	S	A	Y	Y	R	G	-	H	G	H	T	S	T	H
HN101		T	C	G	A	T	C	G	G	C	G	T	G	T	C	T	G
HN054		T	C	G	A	T	C	G	G	C	G	T	G	T	C	T	G
HN103		T	C	G	A	T	C	G	G	C	G	T	G	T	C	T	G
HN102		T	C	G	A	T	C	G	G	C	G	T	G	T	C	T	G
HN088		T	C	G	A	T	C	G	G	C	G	T	G	T	C	T	G
HN093		T	C	G	A	T	C	G	G	C	G	T	G	T	C	T	G
HN065		T	C	G	A	T	C	G	G	C	G	T	G	T	C	T	G
HN030		T	C	G	A	T	C	G	G	C	G	T	G	T	C	T	G
HN022		A	A	C	A	C	T	A	A	T	A	T	A	T	G	A	T
HN039		H	C	G	A	H	C	G	G	C	G	T	R	T	C	H	G
HN040		A	C	G	A	C	C	G	G	C	G	G	A	T	G	A	G
HN002		A	A	C	A	C	T	A	A	T	A	G	A	T	G	A	T
HN008	SV-2 (T)	A	A	C	A	C	T	A	A	T	A	G	A	T	G	A	T
HN009		A	A	C	A	C	T	A	A	T	A	G	A	T	G	A	T
HN010		A	A	C	A	C	T	A	A	T	A	G	A	T	G	A	T

HN013		A	A	C	A	C	T	A	A	T	A	G	A	T	G	A	T
HN015		A	A	C	A	C	T	A	A	T	A	G	A	T	G	A	T
HN018		A	A	C	A	C	T	A	A	T	A	G	A	T	G	A	T
HN020		A	A	C	A	C	T	A	G	C	A	G	A	T	G	A	T
HN021		A	A	C	A	C	T	A	A	T	A	G	A	T	G	A	T
HN024		A	A	C	A	C	T	A	A	T	A	G	A	T	G	A	T
HN025		A	A	C	A	C	T	A	G	T	A	G	A	T	G	A	T
HN027		T	A	C	A	C	T	A	-	C	A	T	A	T	G	A	T
HN028		A	A	C	A	C	T	A	A	T	A	G	A	T	G	A	T
HN034		A	A	C	A	C	T	A	A	T	A	G	A	T	G	A	T
HN047		A	A	C	A	C	T	A	A	T	A	G	A	T	G	A	T
HN055		A	A	C	A	C	T	A	A	T	A	G	A	T	G	A	T
HN056		A	A	C	A	C	T	A	A	T	A	G	A	T	G	A	T
HN059		A	A	C	A	C	T	A	A	T	A	G	A	T	G	A	T
HN060		A	A	C	A	C	T	A	A	T	A	G	A	T	G	A	T
HN063		A	A	C	A	C	T	A	A	T	A	G	A	T	G	A	T
HN069		A	A	C	A	C	T	A	A	T	A	G	A	T	G	A	T
HN074		A	A	C	A	C	T	A	A	T	A	G	A	T	G	A	T
HN075		A	A	C	A	C	T	A	A	T	A	G	A	T	G	A	T
HN076		A	A	C	A	C	T	A	A	T	A	G	A	T	G	A	T
HN077		A	A	C	A	C	T	A	A	T	A	G	A	T	G	A	T
HN079		A	A	C	A	C	T	A	A	T	A	G	A	T	G	A	T
HN080		A	A	C	A	C	T	A	A	T	A	G	A	T	G	A	T
HN082		A	A	C	A	C	T	A	A	T	A	G	A	T	G	A	T
HN084		A	A	C	A	C	T	A	A	T	A	T	A	T	G	A	T
HN085		A	A	C	A	C	T	A	A	T	A	G	A	T	G	A	T
HN086		A	A	C	A	C	T	A	A	-	A	G	A	T	G	A	T
HN016		A	A	C	A	C	T	A	A	T	A	G	A	T	G	A	T
HN067		A	A	C	A	C	T	A	A	T	A	G	A	T	G	A	T
HN090		A	A	C	A	C	T	A	A	T	A	G	A	T	G	A	T
HN105		A	A	C	A	C	T	A	A	T	A	G	A	T	G	A	T
HN106		A	A	C	A	C	T	A	A	T	A	G	A	T	G	A	T
HN057		T	C	G	H	T	C	G	G	C	G	T	G	H	C	T	G
HN038	SV-3 (S)	T	C	G	T	-	C	G	G	C	G	T	-	C	C	A	G
HN001		T	C	G	T	-	-	-	G	C	G	T	-	C	C	A	G

HN006	T	C	G	T	-	C	-	G	C	G	T	-	C	C	A	G
HN007	T	C	G	T	-	-	G	G	C	G	T	-	C	C	A	G
HN012	T	C	G	T	-	-	-	G	C	G	T	-	C	C	A	G
HN023	T	C	G	T	-	-	G	G	C	G	T	-	C	C	A	G
HN026	T	C	G	T	-	-	G	G	C	G	T	-	C	C	A	G
HN031	T	C	G	T	-	-	-	G	C	G	T	-	C	C	A	G
HN032	T	C	G	T	-	-	G	G	C	G	T	-	C	C	A	G
HN033	T	C	G	T	-	-	G	G	C	G	T	-	C	C	A	G
HN035	T	C	G	T	-	-	G	G	C	G	T	-	C	C	A	G
HN041	T	C	G	T	-	-	G	G	C	G	T	-	C	C	A	G
HN043	T	C	G	T	-	C	G	G	C	G	T	-	C	C	A	G
HN044	T	C	G	T	-	-	G	G	C	G	T	-	C	C	A	G
HN045	T	C	G	T	-	-	G	G	C	G	T	-	C	C	A	G
HN046	T	C	G	T	-	-	G	G	C	G	T	-	C	C	A	G
HN048	T	C	G	T	-	-	G	G	C	G	T	-	C	C	A	G
HN052	T	C	G	T	-	-	G	G	C	G	T	-	C	C	A	G
HN053	T	C	G	T	-	-	G	G	C	G	T	-	C	C	A	G
HN058	T	H	H	A	C	T	A	H	H	H	H	A	H	S	A	H
HN061	T	C	G	T	-	-	G	G	C	G	T	-	C	C	A	G
HN064	T	C	G	T	-	C	G	G	C	G	T	-	C	C	A	G
HN068	T	C	G	T	-	C	G	G	C	G	T	-	C	C	A	G
HN070	T	C	G	T	-	-	-	G	C	G	T	-	C	C	A	G
HN071	T	C	G	T	-	-	G	G	C	G	T	-	C	C	A	G
HN073	T	C	G	A	-	T	G	G	C	G	T	-	C	C	A	G
HN100	T	C	G	T	-	-	G	G	C	G	T	-	C	C	A	G
HN083	T	C	G	T	-	-	G	G	C	G	T	-	C	C	A	G
HN091	T	C	G	T	-	-	G	G	C	G	T	-	C	C	A	G
HN094	T	C	G	T	-	C	G	G	C	G	T	-	C	C	A	G
HN096	T	C	G	T	-	-	G	G	C	G	T	-	C	C	A	G
HN097	T	C	G	T	-	C	G	G	C	G	T	-	C	C	T	G
HN036	T	C	G	T	-	-	-	G	C	-	T	-	C	C	A	G
HN098	T	C	G	T	-	C	G	G	C	G	T	-	C	C	A	G
HN050	T	C	G	T	-	C	G	G	C	G	T	-	C	C	A	G
HN095	T	C	G	T	-	-	G	G	C	G	T	-	C	C	A	G

HN104	T	C	G	T	-	C	G	G	C	G	T	-	C	C	A	G
HN107	T	C	G	T	-	-	-	G	C	G	T	-	C	C	A	G
HN037	T	C	G	T	-	C	G	G	C	G	T	-	C	C	A	G
HN108	T	C	G	T	-	C	G	G	C	G	T	-	C	C	A	G
HN078	T	C	G	T	-	-	G	G	C	G	T	-	C	C	A	G

Note : SNPs denoted as M1-M6 were used to develop KASPar assay. The marker M1, M4 and M6 (highlighted with red text) are associated with tolerant and sensitive group. "H" represents heterozygote.

Supplementary Table S4: Validation of six KASPar assay using 104 resequencing lines and correlation between genotype and phenotype data.

Seq Id	SV	M1*	Genotype vs Phenotype	M2	Genotype vs SV	M3	Genotype vs SV	M4*	Genotype vs Phenotype	M5	Genotype vs SV	M6*	Genotype vs Phenotype
HN011	SV-1	G	Matched	A	Matched	T	Matched	C	Matched	T	Matched	G	Matched
HN014	SV-1	G	Matched	A	Matched	T	Matched	C	Matched	T	Matched	G	Matched
HN029	SV-1	G	Matched	A	Matched	T	Matched	C	Matched	T	Matched	G	Matched
HN030	SV-1	G	Matched	A	Matched	T	Matched	C	Matched	T	Matched	G	Matched
HN039	SV-1	G	Not Matched	A	Matched	T	Matched	C	Not Matched	H	Matched	G	Not Matched
HN042	SV-1	G	Matched	A	Matched	T	Matched	C	Matched	T	Matched	G	Matched
HN049	SV-1	G	Matched	A	Matched	T	Matched	C	Matched	T	Matched	G	Matched
HN051	SV-1	G	Matched	A	Matched	T	Matched	C	Matched	T	Matched	G	Matched
HN054	SV-1	G	Matched	A	Matched	T	Matched	C	Matched	T	Matched	G	Matched
HN057	SV-1	G	Matched	H	Matched	H	Matched	H	Matched	H	Matched	G	Matched
HN058	SV-1	H	Matched	H	Matched	Y	Matched	H	Matched	A	Not Matched	T	Matched
HN062	SV-1	G	Matched	A	Matched	T	Matched	C	Matched	T	Matched	G	Matched
HN065	SV-1	G	Matched	A	Matched	T	Matched	C	Matched	T	Matched	G	Matched
HN066	SV-1	G	Matched	A	Matched	T	Matched	C	Matched	T	Matched	G	Matched
HN081	SV-1	H	Matched	A	Matched	T	Matched	C	Matched	T	Matched	H	Matched
HN088	SV-1	G	Not Matched	A	Matched	T	Matched	C	Not Matched	T	Matched	G	Not Matched
HN089	SV-1	G	Matched	A	Matched	T	Matched	C	Matched	T	Matched	G	Matched
HN092	SV-1	G	Matched	A	Matched	T	Matched	C	Matched	T	Matched	G	Matched
HN099	SV-1	G	Matched	A	Matched	T	Matched	C	Matched	T	Matched	G	Matched
HN101	SV-1	G	Not Matched	A	Matched	T	Matched	C	Not Matched	T	Matched	G	Not Matched
HN102	SV-1	G	Matched	A	Matched	T	Matched	C	Matched	T	Matched	G	Matched
HN103	SV-1	G	Matched	A	Matched	T	Matched	C	Matched	T	Matched	G	Matched
HN008	SV-2	C	Matched	A	Matched	T	Matched	G	Matched	A	Matched	T	Matched
HN009	SV-2	C	Matched	A	Matched	T	Matched	G	Matched	A	Matched	T	Matched
HN010	SV-2	C	Matched	A	Matched	T	Matched	G	Matched	A	Matched	T	Matched
HN013	SV-2	C	Matched	A	Matched	T	Matched	G	Matched	A	Matched	T	Matched
HN015	SV-2	C	Matched	A	Matched	T	Matched	G	Matched	A	Matched	T	Matched
HN016	SV-2	C	Not Matched	A	Matched	T	Matched	G	Not Matched	A	Matched	T	Not Matched
HN017	SV-2	C	Not Matched	A	Matched	T	Matched	G	Not Matched	A	Matched	T	Not Matched
HN018	SV-2	C	Not Matched	A	Matched	T	Matched	G	Not Matched	A	Matched	T	Not Matched
HN020	SV-2	C	Not Matched	A	Matched	T	Matched	G	Not Matched	A	Matched	T	Not Matched

HN021	SV-2	C	Matched	A	Matched	T	Matched	G	Matched	A	Matched	T	Matched
HN022	SV-2	C	Not Matched	A	Matched	T	Matched	G	Not Matched	A	Not Matched	T	Not Matched
HN024	SV-2	C	Matched	A	Matched	T	Matched	G	Matched	A	Matched	T	Matched
HN025	SV-2	C	Matched	A	Matched	T	Matched	G	Matched	A	Matched	T	Matched
HN027	SV-2	C	Matched	A	Matched	T	Matched	G	Matched	A	Matched	T	Matched
**HN028**	SV-2	C	Matched	A	Matched	T	Matched	G	Matched	A	Matched	T	Matched
HN040	SV-2	G	Not Matched	A	Matched	T	Matched	G	Matched	A	Matched	G	Not Matched
HN047	SV-2	C	Matched	A	Matched	T	Matched	G	Matched	A	Matched	T	Matched
HN055	SV-2	C	Matched	A	Matched	T	Matched	G	Matched	A	Matched	T	Matched
HN056	SV-2	C	Matched	A	Matched	T	Matched	G	Matched	A	Matched	T	Matched
HN060	SV-2	C	Matched	A	Matched	T	Matched	G	Matched	A	Matched	T	Matched
**HN063**	SV-2	C	Matched	A	Matched	T	Matched	G	Matched	A	Matched	T	Matched
HN067	SV-2	C	Matched	A	Matched	T	Matched	G	Matched	A	Matched	T	Matched
HN069	SV-2	C	Matched	A	Matched	T	Matched	G	Matched	A	Matched	T	Matched
HN074	SV-2	C	Matched	A	Matched	T	Matched	G	Matched	A	Matched	T	Matched
HN075	SV-2	C	Matched	A	Matched	T	Matched	G	Matched	A	Matched	T	Matched
HN076	SV-2	C	Matched	A	Matched	T	Matched	G	Matched	A	Matched	T	Matched
HN077	SV-2	C	Matched	A	Matched	T	Matched	G	Matched	A	Matched	T	Matched
HN079	SV-2	C	Matched	A	Matched	T	Matched	G	Matched	A	Matched	T	Matched
HN080	SV-2	C	Matched	A	Matched	T	Matched	G	Matched	A	Matched	T	Matched
HN082	SV-2	C	Matched	A	Matched	T	Matched	G	Matched	A	Matched	T	Matched
HN084	SV-2	C	Matched	A	Matched	T	Matched	G	Matched	A	Matched	T	Matched
HN085	SV-2	C	Matched	A	Matched	T	Matched	G	Matched	A	Matched	T	Matched
HN086	SV-2	C	Matched	A	Matched	T	Matched	G	Matched	A	Matched	T	Matched
HN087	SV-2	G	Matched	A	Matched	T	Matched	C	Matched	T	Not Matched	G	Matched
HN090	SV-2	C	Matched	A	Matched	T	Matched	G	Matched	A	Matched	T	Matched
**HN105**	SV-2	C	Matched	A	Matched	T	Matched	G	Matched	A	Matched	T	Matched
HN106	SV-2	C	Not Matched	A	Matched	T	Matched	G	Not Matched	A	Matched	T	Not Matched
HN002	SV-2	C	Not Matched	A	Matched	T	Matched	G	Not Matched	A	Matched	T	Not Matched
HN003	SV-2	G	Matched	A	Matched	T	Matched	C	Matched	A	Matched	G	Matched
HN004	SV-2	G	Matched	A	Matched	T	Matched	C	Matched	A	Matched	G	Matched
HN005	SV-2	G	Matched	A	Matched	T	Matched	C	Matched	A	Matched	G	Matched
HN072	SV-2	G	Matched	T	Not Matched	T	Matched	C	Matched	A	Matched	G	Matched
HN006	SV-3	G	Matched	T	Matched	C	Matched	C	Matched	A	Matched	G	Matched
HN007	SV-3	G	Matched	T	Matched	C	Matched	C	Matched	A	Matched	G	Matched
HN012	SV-3	G	Matched	T	Matched	C	Matched	C	Matched	A	Matched	G	Matched

HN023	SV-3	G	Matched	T	Matched	C	Matched	C	Matched	A	Matched	G	Matched
HN026	SV-3	G	Matched	T	Matched	C	Matched	C	Matched	A	Matched	G	Matched
HN031	SV-3	G	Matched	T	Matched	C	Matched	C	Matched	A	Matched	G	Matched
HN032	SV-3	G	Matched	T	Matched	C	Matched	C	Matched	A	Matched	G	Matched
HN033	SV-3	G	Matched	T	Matched	C	Matched	C	Matched	A	Matched	G	Matched
HN035	SV-3	G	Matched	T	Matched	C	Matched	C	Matched	A	Matched	G	Matched
HN036	SV-3	G	Matched	T	Matched	C	Matched	C	Matched	A	Matched	G	Matched
HN037	SV-3	G	Matched	T	Matched	C	Matched	C	Matched	A	Matched	G	Matched
HN038	SV-3	G	Matched	T	Matched	C	Matched	C	Matched	A	Matched	G	Matched
HN041	SV-3	G	Matched	T	Matched	C	Matched	C	Matched	A	Matched	G	Matched
HN043	SV-3	G	Matched	T	Matched	C	Matched	C	Matched	A	Matched	G	Matched
HN044	SV-3	G	Matched	T	Matched	C	Matched	C	Matched	A	Matched	G	Matched
HN045	SV-3	G	Matched	T	Matched	C	Matched	C	Matched	A	Matched	G	Matched
HN046	SV-3	G	Matched	T	Matched	C	Matched	C	Matched	A	Matched	G	Matched
HN048	SV-3	G	Matched	T	Matched	C	Matched	C	Matched	A	Matched	G	Matched
HN050	SV-3	G	Matched	T	Matched	C	Not Matched	C	Matched	A	Not Matched	G	Matched
HN052	SV-3	G	Matched	T	Matched	C	Matched	C	Matched	A	Matched	G	Matched
HN053	SV-3	G	Matched	T	Matched	C	Matched	C	Matched	A	Matched	G	Matched
HN061	SV-3	G	Matched	T	Matched	C	Matched	C	Matched	A	Matched	G	Matched
HN064	SV-3	G	Matched	T	Matched	C	Matched	C	Matched	A	Matched	G	Matched
HN068	SV-3	G	Matched	T	Matched	C	Matched	C	Matched	A	Matched	G	Matched
HN070	SV-3	G	Matched	T	Matched	C	Matched	C	Matched	A	Matched	G	Matched
HN071	SV-3	G	Matched	T	Matched	C	Matched	C	Matched	A	Matched	G	Matched
HN073	SV-3	G	Matched	A	Not Matched	C	Matched	C	Matched	A	Matched	G	Matched
HN078	SV-3	G	Matched	T	Matched	C	Matched	C	Matched	A	Matched	G	Matched
HN083	SV-3	G	Matched	T	Matched	C	Matched	C	Matched	A	Matched	G	Matched
HN091	SV-3	G	Matched	T	Matched	C	Matched	C	Matched	A	Not Matched	G	Matched
HN094	SV-3	G	Matched	t	Matched	C	Matched	C	Matched	A	Matched	G	Matched
HN095	SV-3	G	Matched	t	Matched	C	Matched	C	Matched	A	Matched	G	Matched
HN096	SV-3	G	Matched	T	Matched	C	Matched	C	Matched	A	Matched	G	Matched
HN097	SV-3	G	Matched	T	Matched	C	Matched	C	Matched	T	Not Matched	G	Matched
HN098	SV-3	G	Matched	T	Matched	C	Matched	C	Matched	A	Matched	G	Matched
HN100	SV-3	G	Matched	T	Matched	C	Matched	C	Matched	A	Matched	G	Matched
HN104	SV-3	G	Matched	T	Matched	C	Matched	C	Matched	A	Matched	G	Matched
HN107	SV-3	G	Matched	T	Matched	C	Matched	C	Matched	A	Matched	G	Matched
**HN001**	SV-3	G	Matched	T	Matched	C	Matched	C	Matched	A	Matched	G	Matched

Reference	SV-1	G	Matched	A	Matched	T	Matched	C	Matched	A	Matched	G	Matched

*Note: * denotes these marker can differentiate salt-tolerant and salt-sensitive lines. Highlighted sequence id represents lines with known salinity reaction. Accession with underline represents wild soybean lines. "H" represents heterozygote.*

Supplementary Table S5. Genotypic evaluation of F_8 RIL population derived from (PI 483463 x Hutcheson) using M6 KASPar assay. Artificial Het - W82:HN063 (1:1 ratio).

RIL	LSS	Genotype call	Genotype vs Phenotype
**W82**	4.3	WT	Matched
**Artificial HET**	-	HET	Matched
**HN063**	1	MUT	Matched
**H2O**	**-**	**-**	-
RIL4	4.1	WT	Matched
RIL5	1.4	MUT	Matched
RIL6	2.1	MUT	Matched
RIL9	1.5	MUT	Matched
RIL12	1.6	MUT	Matched
RIL14	1.4	MUT	Matched
RIL15	2.4	MUT	Matched
RIL18	1.1	WT	Not Matched
RIL19	2.1	MUT	Matched
RIL23	4.4	WT	Matched
RIL25	3.5	WT	Matched
RIL26	2.9	WT	Matched
RIL28	3.2	WT	Matched
RIL29	3.0	WT	Matched
RIL30	1.1	MUT	Matched
RIL31	1.3	MUT	Matched
RIL32	2.0	MUT	Matched
RIL34	1.9	MUT	Matched
RIL35	2.0	HET	Matched
RIL36	1.6	MUT	Matched
RIL37	3.7	WT	Matched
RIL39	1.3	MUT	Matched
RIL41	1.7	WT	Not Matched
RIL43	1.1	MUT	Matched
RIL46	1.1	MUT	Matched
RIL48	4.2	WT	Matched
RIL49	3.0	WT	Matched

RIL51	1.5	MUT	Matched
RIL53	2.2	WT	Not Matched
RIL56	4.0	WT	Matched
RIL58	4.2	WT	Matched
RIL59	4.1	WT	Matched
RIL60	1.2	MUT	Matched
RIL61	1.7	MUT	Matched
RIL62	3.6	WT	Matched
RIL63	3.0	WT	Matched
RIL64	1.9	WT	Not Matched
RIL65	3.6	WT	Matched
RIL66	1.7	MUT	Matched
RIL67	1.5	MUT	Matched
RIL70	1.4	MUT	Matched
RIL72	1.6	MUT	Matched
RIL74	3.9	WT	Matched
RIL76	4.1	WT	Matched
RIL77	3.8	WT	Matched
RIL79	2.8	WT	Matched
RIL86	2.4	WT	Not Matched
RIL87	1.3	MUT	Matched
RIL90	3.0	WT	Matched
RIL91	1.8	MUT	Matched
RIL94	2.5	MUT	Matched
RIL97	1.3	MUT	Matched
RIL98	4.0	WT	Matched
RIL101	3.4	WT	Matched
RIL102	3.2	WT	Matched
RIL110	3.2	WT	Matched
RIL113	3.1	WT	Matched
RIL122	3.9	WT	Matched
RIL127	3.0	WT	Matched
RIL129	2.9	WT	Matched
RIL141	3.7	WT	Matched
RIL143	3.3	WT	Matched
RIL147	1.4	MUT	Matched

RIL149	3.6	WT	Matched
RIL154	3.2	WT	Matched
RIL157	2.3	HET	Matched
RIL160	3.5	WT	Matched
RIL161	1.6	MUT	Matched
RIL163	3.9	WT	Matched
RIL168	1.3	MUT	Matched
RIL169	3.6	WT	Matched
RIL170	4.0	WT	Matched
RIL172	3.8	WT	Matched
RIL173	2.9	MUT	Matched
RIL176	3.2	WT	Matched
RIL181	3.5	WT	Matched
RIL182	1.8	MUT	Matched
RIL184	2.2	MUT	Matched
RIL188	1.3	MUT	Matched
RIL189	3.0	WT	Matched
RIL191	1.7	MUT	Matched
RIL192	3.2	WT	Matched
RIL193	3.2	WT	Matched
RIL196	3.3	WT	Matched
RIL199	4.3	WT	Matched
RIL200	2.8	MUT	Matched
RIL207	3.0	WT	Matched
RIL216	3.2	WT	Matched
RIL218	2.8	WT	Matched
RIL228	3.3	MUT	Matched
**HN063 (PI 483463)**	1.0	MUT	Matched
**HN001 (Hutcheson)**	4.8	WT	Matched

Supplementary Table S6: KASPar assay primer sequence information. Marker allele labelled with FAM (wild-type) and HEX (mutant type) fluorescence dye.

Marker	Fluroscent primer	Sequence
M1	FAM_primer	ACCAAACCAAACCTAGCTAGTTTTCATCACCTTCCTATGATTGTTTTTGTTTTAATTTCCTAACTAACTAACACAGCAA**G**
	HEX_primer	ACCAAACCAAACCTAGCTAGTTTTCATCACCTTCCTATGATTGTTTTTGTTTTAATTTCCTAACTAACTAACACAGCAA**C**
	Common Reverse primer	AAGCACTGAGTCTTTGGCCATGACGTTCAACGCGAGCACCATCACAACGGCGTCGGA
M2	FAM_primer	AAGCACTGAGTCTTTGGCCATGACGTTCAACGCGAGCACCATCACAACGGCGTCGGA**A**
	HEX_primer	AAGCACTGAGTCTTTGGCCATGACGTTCAACGCGAGCACCATCACAACGGCGTCGGA**T**
	Common Reverse primer	GGAGCCTGGCAGGGCGATAATCC**Y**CTGAACCACGCTCTTCCTTTGTTGATCGTTCAAACCATCCTCGTAGTCTTCG
M3	FAM_primer	TAATCGTTCTCAATATTGGCAGAGAGAAGAAGGTGAGTTATGCTTTACATTGCTATTGATGTAGTAGTAA**T**
	HEX_primer	TAATCGTTCTCAATATTGGCAGAGAGAAGAAGGTGAGTTATGCTTTACATTGCTATTGATGTAGTAGTAA**C**
	Common Reverse primer	TATACAAATATGTTGTTTTGTGTTATTCTACGATTATCATCATCTAGTT
M4	FAM_primer	TATACAAATATGTTGTTTTGTGTTATTCTACGATTATCATCATCTAGTT**C**
	HEX_primer	TATACAAATATGTTGTTTTGTGTTATTCTACGATTATCATCATCTAGTT**G**
	Common Reverse primer	AATGGTTAACTTTATCACATATTTATAGTTGAATATAAGTTAGTTTATTAATTTTCAACTAAAAATTAAC
M5	FAM_primer	GGGGTTGCCAGAAGGTACGAACAAGAACCTGAGACAAGTGTTGCTGCAAGGAAAAGAGTGTGCATA**T**
	HEX_primer	GGGGTTGCCAGAAGGTACGAACAAGAACCTGAGACAAGTGTTGCTGCAAGGAAAAGAGTGTGCATA**A**
	Common Reverse primer	TTTTCATTGGTGGACCACATGATCGCAAGGTTTTGGAGTTAGGTAGCAGAATGGCAGAGCATCCAGCAATTAGG
M6	FAM_primer	GAGATCTCTTTGTCTCTTCGGGTAACGGCATTACCAGTTCATTGCTCGTTATACAGGACCGATATTT**G**
	HEX_primer	GAGATCTCTTTGTCTCTTCGGGTAACGGCATTACCAGTTCATTGCTCGTTATACAGGACCGATATTT**T**
	Common Reverse primer	ATAAATTCAAACGAAAGTAATCTCGTTAAGACATCAAGGGCCGAGAGTACTGTGATTAAAGATGCTATCGAAGAACTTTAA

Supplementary Figure S1: Effect of salt treatment on accumulation of Na^+ in soybean leaf tissue under normal (0 mM) and salt-treatment (120 mM NaCl) conditions. Tolerant (T); Sensitive (S).

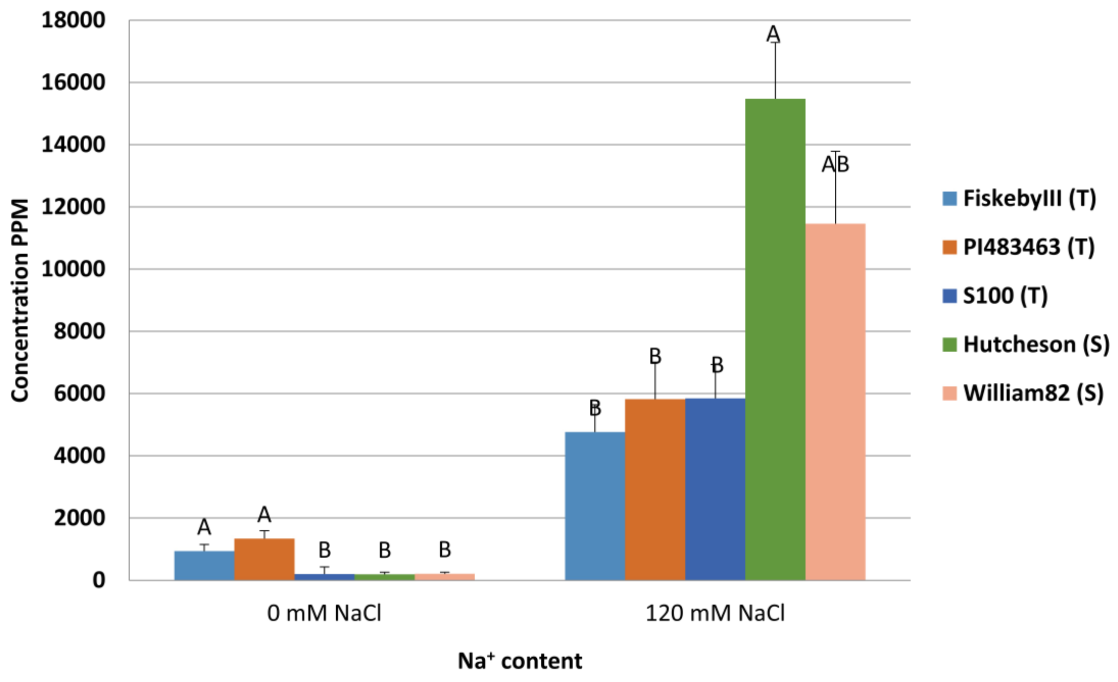

Supplementary Figure S2: Manhattan plots of GWAS for **(A)** LSS and, **(B)** SPAD ratio, in 106 soybean lines using SoySNP50K dataset. Negative $\log_{10}$-trransformed *P* values of SNPs from genome-wide scan for salinity traits using EMMAX model including kinship and population structure are plotted against positions on each of the 20 chromosomes; **(C)** genes underlying significant trait-associated SNPs on Chr. 3.

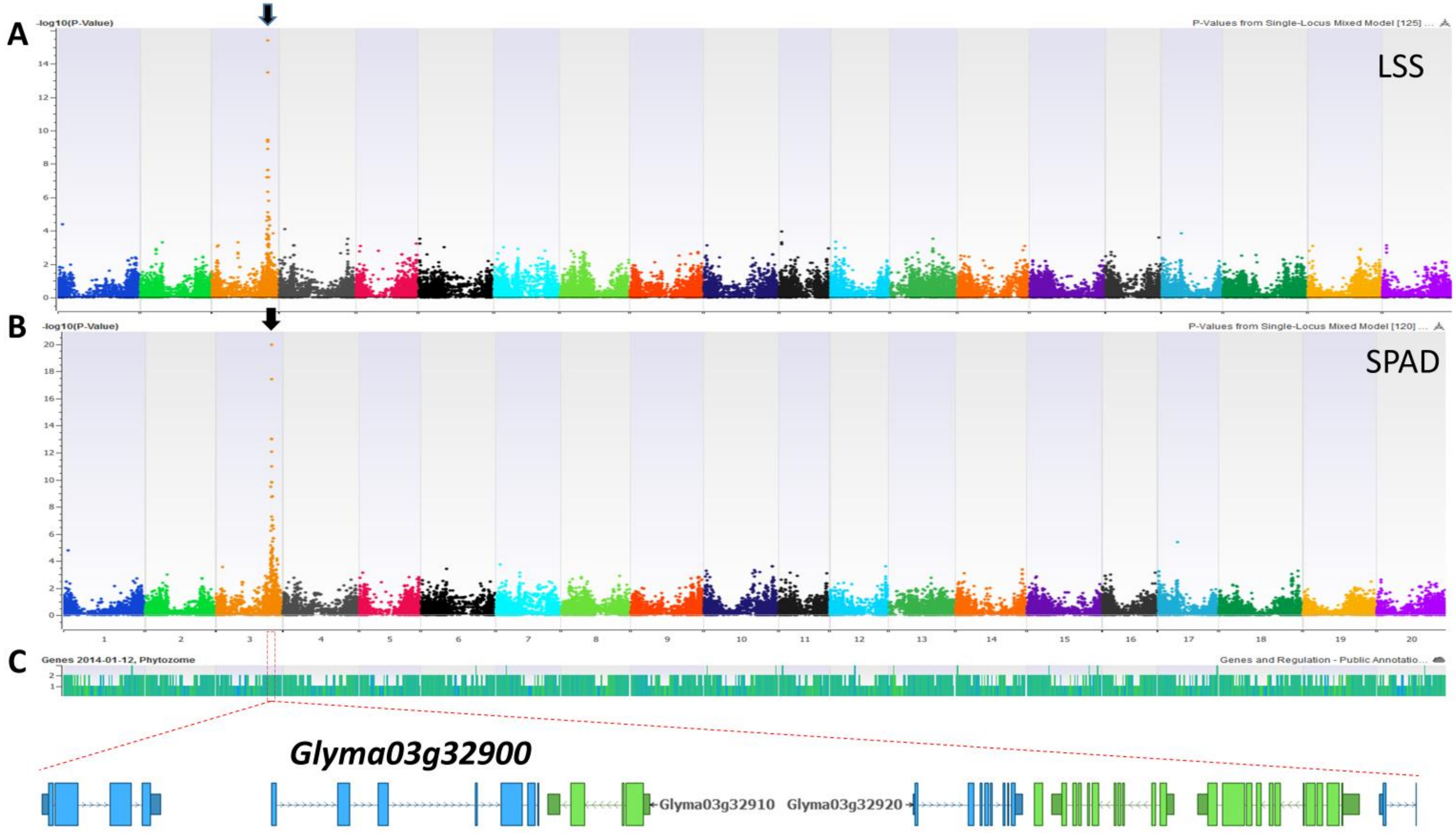

Supplementary Figure S3: Comparison of SNPs between SV-2 and SV-3 in promoter and coding regions of the gene *GmCHX1*.

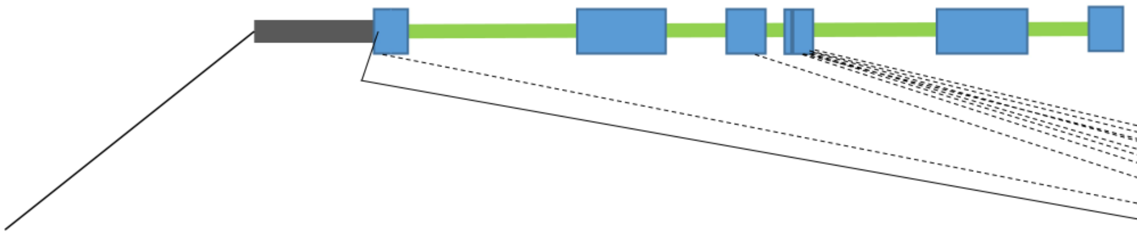

Position from start codon (bp)

	-1989	-1942	-1939	-1937	-1931	-1865	-1807	-1639	-1549	-1520	-1498	-1457	-1429	-1409	-1385
SV- 2 (Tolerant)	T	A	C	A	C	A	C	A	A	A	G	C	G	G	A
	T	A	C	A	C	A	C	A	A	A	G	C	G	G	A
SV- 3 (Sensitive)	C	G	T	T	T	G	G	C	C	C	A	G	A	T	G
	C	G	T	T	T	G	G	C	C	C	A	G	A	T	G

	-1334	-1287	-1276	-1103	-1101	-1078	-884	-641	-541	-446	-434	-415	-356	-290
SV- 2 (Tolerant)	T	T	T	C	C	A	T	G	G	G	C	A	C	A
	T	T	T	C	C	A	T	G	G	G	C	A	C	A
SV- 3 (Sensitive)	A	G	G	T	T	G	C	C	A	A	A	G	G	T
	A	G	G	T	T	G	C	C	A	A	A	G	G	T

AA position

	13	354	412	450	516	591	603	606	703
SV- 2 (Tolerant)	E	S	L	E	M	L	A	V	V
	E	S	L	E	M	L	A	V	V
SV- 3 (Sensitive)	D	C	V	G	I	W	P	I	A
	D	C	L	G	M	W	P	V	V

Supplementary Figure S4: Haplotype analysis of 106 and 23 lines[42] WGRS data. Base position identical to the reference (W82) are white, black – different, gray-missing data.

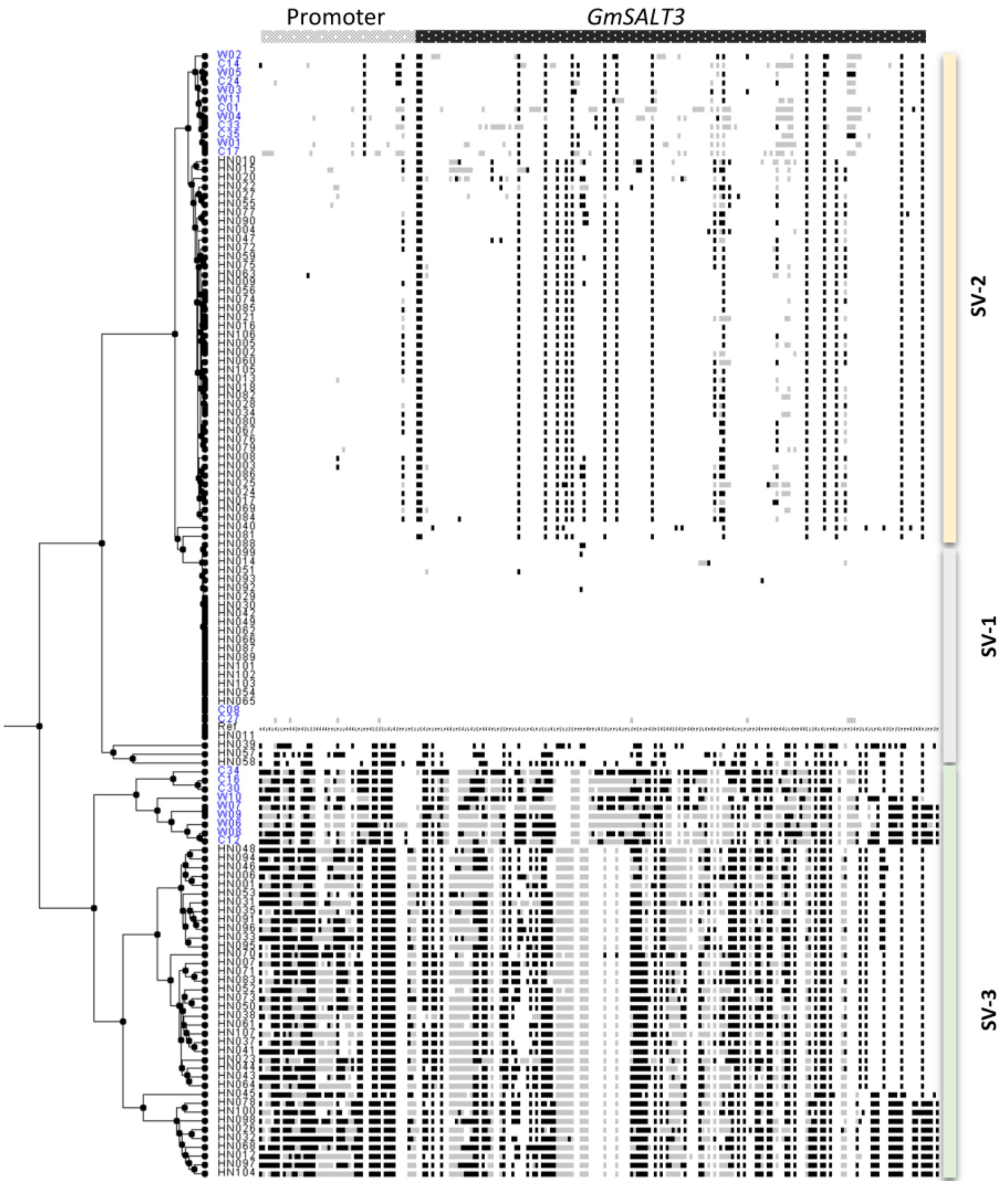

Supplementary Figure S5: GmCHX1 protein transmembrane helix prediction obtained using TMHMM 2.0 tool[78]. Arrow denotes deletion.

```
# WEBSEQUENCE Length: 811
# WEBSEQUENCE Number of predicted TMHs:  10
# WEBSEQUENCE Exp number of AAs in TMHs: 241.59121
# WEBSEQUENCE Exp number, first 60 AAs:  27.09195
# WEBSEQUENCE Total prob of N-in:        0.18346
# WEBSEQUENCE POSSIBLE N-term signal sequence
WEBSEQUENCE     TMHMM2.0        outside      1    24
WEBSEQUENCE     TMHMM2.0        TMhelix     25    47
WEBSEQUENCE     TMHMM2.0        inside      48    53
WEBSEQUENCE     TMHMM2.0        TMhelix     54    71
WEBSEQUENCE     TMHMM2.0        outside     72    85
WEBSEQUENCE     TMHMM2.0        TMhelix     86   108
WEBSEQUENCE     TMHMM2.0        inside     109   119
WEBSEQUENCE     TMHMM2.0        TMhelix    120   142
WEBSEQUENCE     TMHMM2.0        outside    143   156
WEBSEQUENCE     TMHMM2.0        TMhelix    157   179
WEBSEQUENCE     TMHMM2.0        inside     180   191
WEBSEQUENCE     TMHMM2.0        TMhelix    192   214
WEBSEQUENCE     TMHMM2.0        outside    215   223   ← ~180 bp deletion in exon 3
WEBSEQUENCE     TMHMM2.0        TMhelix    224   243
WEBSEQUENCE     TMHMM2.0        inside     244   263
WEBSEQUENCE     TMHMM2.0        TMhelix    264   286
WEBSEQUENCE     TMHMM2.0        outside    287   342
WEBSEQUENCE     TMHMM2.0        TMhelix    343   365
WEBSEQUENCE     TMHMM2.0        inside     366   406
WEBSEQUENCE     TMHMM2.0        TMhelix    407   429
WEBSEQUENCE     TMHMM2.0        outside    430   811
```

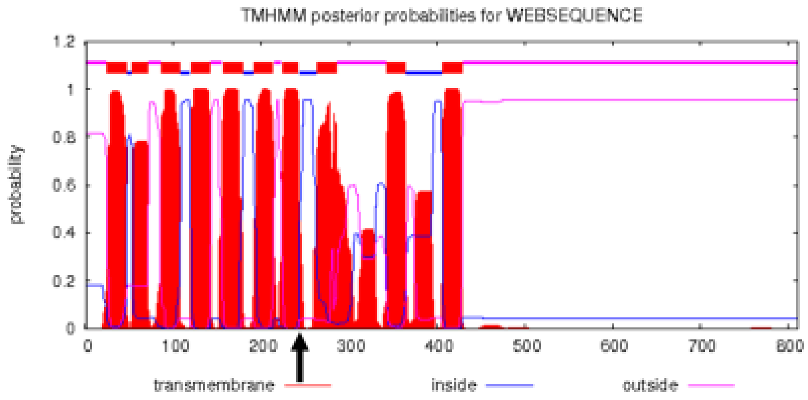

plot in postscript, script for making the plot in gnuplot, data for plot
